# The Germline and Somatic Origins of Prostate Cancer Heterogeneity

**DOI:** 10.1158/2159-8290.CD-23-0882

**Published:** 2025-02-13

**Authors:** Takafumi N. Yamaguchi, Kathleen E. Houlahan, Helen Zhu, Natalie Kurganovs, Julie Livingstone, Natalie S. Fox, Jiapei Yuan, Jocelyn Sietsma Penington, Chol-Hee Jung, Tommer Schwarz, Weerachai Jaratlerdsiri, Job van Riet, Peter Georgeson, Stefano Mangiola, Kodi Taraszka, Robert Lesurf, Jue Jiang, Ken Chow, Lawrence E. Heisler, Yu-Jia Shiah, Susmita G. Ramanand, Michael J. Clarkson, Anne Nguyen, Shadrielle Melijah G. Espiritu, Ryan Stuchbery, Richard Jovelin, Vincent Huang, Connor Bell, Edward O’Connor, Patrick J. McCoy, Christopher M. Lalansingh, Marek Cmero, Adriana Salcedo, Eva K.F. Chan, Lydia Y. Liu, Phillip D. Stricker, Vinayak Bhandari, Riana M.S. Bornman, Dorota H.S. Sendorek, Andrew Lonie, Stephenie D. Prokopec, Michael Fraser, Justin S. Peters, Adrien Foucal, Shingai B.A. Mutambirwa, Lachlan Mcintosh, Michèle Orain, Matthew Wakefield, Valérie Picard, Daniel J. Park, Hélène Hovington, Michael Kerger, Alain Bergeron, Veronica Sabelnykova, Ji-Heui Seo, Mark M. Pomerantz, Noah Zaitlen, Sebastian M. Waszak, Alexander Gusev, Louis Lacombe, Yves Fradet, Andrew Ryan, Amar U. Kishan, Martijn P. Lolkema, Joachim Weischenfeldt, Bernard Têtu, Anthony J. Costello, Vanessa M. Hayes, Rayjean J. Hung, Housheng H. He, John D. McPherson, Bogdan Pasaniuc, Theodorus van der Kwast, Anthony T. Papenfuss, Matthew L. Freedman, Bernard J. Pope, Robert G. Bristow, Ram S. Mani, Niall M. Corcoran, Jüri Reimand, Christopher M. Hovens, Paul C. Boutros

**Affiliations:** 1 Ontario Institute for Cancer Research, Toronto, Canada.; 2 Department of Human Genetics, University of California, Los Angeles, Los Angeles, California.; 3 Jonsson Comprehensive Cancer Centre, University of California, Los Angeles, Los Angeles, California.; 4 Institute for Precision Health, University of California, Los Angeles, Los Angeles, California.; 5 Department of Medical Biophysics, University of Toronto, Toronto, Canada.; 6 Vector Institute, Toronto, Canada.; 7 Stanford Cancer Institute, Stanford University School of Medicine, Stanford, California.; 8 Princess Margaret Cancer Centre, University Health Network, Toronto, Canada.; 9 Australian Prostate Cancer Research Centre Epworth, Richmond, Australia.; 10 Department of Surgery, The University of Melbourne, Parkville, Australia.; 11 Department of Pathology, UT Southwestern Medical Center, Dallas, Texas.; 12 Bioinformatics Division, Walter and Eliza Hall Institute, Parkville, Australia.; 13 Melbourne Bioinformatics, The University of Melbourne, Melbourne, Australia.; 14 Bioinformatics Interdepartmental Program, University of California, Los Angeles, Los Angeles, California.; 15 Department of Pathology and Laboratory Medicine, University of California, Los Angeles, Los Angeles, California.; 16 Laboratory for Human Comparative and Prostate Cancer Genomics, Genomics and Epigenetics Division, Garvan Institute of Medical Research, Darlinghurst, Australia.; 17 Department of Medical Oncology, Erasmus University, Rotterdam, the Netherlands.; 18 Department of Computer Science, University of California, Los Angeles, Los Angeles, California.; 19 Laboratory for Human Comparative and Prostate Cancer Genomics, Genomics and Epigenetics Theme, Garvan Institute of Medical Research, Darlinghurst, Australia.; 20 Division of Urology, Royal Melbourne Hospital, Parkville, Australia.; 21 Department of Medical Oncology, Dana-Farber Cancer Institute, Boston, Massachusetts.; 22 St Vincent’s Clinical School, University of New South Wales, Randwick, Australia.; 23 Department of Urology, St. Vincent’s Hospital Sydney, Darlinghurst, Australia.; 24 School of Health Systems and Public Health, University of Pretoria, Pretoria, South Africa.; 25 Department of Urology, Sefako Makgatho Health Science University, Medunsa, South Africa.; 26 Research Centre of CHU de Québec-Université Laval, Québec City, Canada.; 27 Division of Urology and Research Centre of CHU de Québec-Université Laval, Québec City, Canada.; 28 Department of Neurology, University of California, Los Angeles, Los Angeles, California.; 29 Department of Computational Medicine, University of California, Los Angeles, Los Angeles, California.; 30 Centre for Molecular Medicine Norway (NCMM), Nordic EMBL Partnership, University of Oslo and Oslo University Hospital, Oslo, Norway.; 31 Swiss Institute for Experimental Cancer Research (ISREC), School of Life Sciences, École Polytechnique Fédérale de Lausanne, Lausanne, Switzerland.; 32 Division of Population Sciences, Dana-Farber Cancer Institute, Boston, Massachusetts.; 33 Division of Genetics, Brigham Women’s Hospital and Harvard Medical School, Boston, Massachusetts.; 34 The Eli and Edythe L. Broad Institute, Cambridge, Massachusetts.; 35 TissuPath Specialist Pathology Services, Mount Waverley, Australia.; 36 Department of Radiation Oncology, University of California, Los Angeles, Los Angeles, California.; 37 Center for Personalized Cancer Treatment, Rotterdam, the Netherlands.; 38 Biotech Research & Innovation Centre (BRIC), University of Copenhagen, Copenhagen, Denmark.; 39 Finsen Laboratory, Rigshospitalet, Copenhagen, Denmark.; 40 Department of Urology, Charité-Universitätsmedizin Berlin, Berlin, Germany.; 41 Central Clinical School, University of Sydney, Camperdown, Australia.; 42 Department of Medical Sciences, University of Limpopo, Mankweng, South Africa.; 43 Prosserman Centre for Population Health Research, Lunenfeld-Tanenbaum Research Institute, Toronto, Canada.; 44 Epidemiology Division, Dalla Lana School of Public Health, University of Toronto, Toronto, Canada.; 45 Department of Medical Biology, University of Melbourne, Parkville, Australia.; 46 Department of Mathematics and Statistics, University of Melbourne, Parkville, Australia.; 47 Peter MacCallum Cancer Centre, Victorian Comprehensive Cancer Centre, Melbourne, Australia.; 48 Sir Peter MacCallum Department of Oncology, University of Melbourne, Parkville, Australia.; 49 Center for Functional Cancer Epigenetics, Dana-Farber Cancer Institute, Boston, Massachusetts.; 50 Department of Clinical Pathology, The University of Melbourne, Parkville, Australia.; 51 Precision Medicine, School of Clinical Sciences at Monash Health, Monash University, Clayton, Australia.; 52 Department of Medicine, Monash University, Clayton, Australia.; 53 Manchester Cancer Research Centre, Manchester, United Kingdom.; 54 Department of Urology, UT Southwestern Medical Center, Dallas, Texas.; 55 Department of Urology, Peninsula Health, Frankston, Australia.; 56 The Victorian Comprehensive Cancer Centre, Parkville, Australia.; 57 Department of Urology, University of California, Los Angeles, Los Angeles, California.

## Abstract

Newly diagnosed prostate cancers differ dramatically in mutational composition and lethality. The most accurate clinical predictor of lethality is tumor tissue architecture, quantified as tumor grade. To interrogate the evolutionary origins of prostate cancer heterogeneity, we analyzed 666 prostate tumor whole genomes. We identified a compendium of 223 recurrently mutated driver regions, most influencing downstream mutational processes and gene expression. We identified and validated individual germline variants that predispose tumors to acquire specific somatic driver mutations: these explain heterogeneity in disease presentation and ancestry differences. High-grade tumors have a superset of the drivers in lower-grade tumors, including increased frequency of *BRCA2* and *MYC* mutations. Grade-associated driver mutations occur early in tumor evolution, and their earlier occurrence strongly predicts cancer relapse and metastasis. Our data suggest high- and low-grade prostate tumors both emerge from a common premalignant field, influenced by germline genomic context and stochastic mutation timing.

**Significance:**

This study uncovered 223 recurrently mutated driver regions using the largest cohort of prostate tumors to date. It reveals associations between germline SNPs, somatic drivers, and tumor aggression, offering significant insights into how prostate tumor evolution is shaped by germline factors and the timing of somatic mutations.

## Teaser

A cohort of 666 localized prostate tumors with whole-genome sequencing is used to quantify how germline and somatic genetic features interact to determine disease evolution and eventual clinical presentation.

## Introduction

Prostate cancer is the most commonly diagnosed internal malignancy in men (1), and increasing life expectancy in the population is sharply increasing its incidence (2). A large fraction of prostate tumors are clinically indolent, not requiring radical treatment (3), yet a subset demonstrates aggressive clinical behavior and metastasizes to distant sites, becoming lethal. Clinicians use clinicopathologic features to distinguish indolent from aggressive tumors, including pretreatment serum concentrations of PSA, tumor grade, and tumor size and extent (T category). Tumor grade is the strongest predictor of localized disease lethality and is determined by expert genitourinary (GU) pathologists by visual inspection of glandular architecture and morphology. Tumors are categorized into five tiers of the International Society of Urological Pathology (ISUP) grade group (GG) system (4), which is a modern update to the well-known Gleason grading system. ISUP GG 1 tumors have minimal metastatic potential, whereas ISUP GG 5 tumors are poorly differentiated with markedly increased risks of dissemination and poor overall prognosis. ISUP grade is central to clinically used prognostic risk-stratification systems like the National Comprehensive Cancer Network (NCCN) guidelines and drives clinical management of patients with localized prostate cancer (5, 6).

It remains unclear how prostate tumors evolve to have different grades. Many molecular features are associated with grade, including mutation density [particularly of copy-number aberrations (CNA)] and mRNA abundance subtypes (7–12). Exome sequencing (13) and meta-analyses (14) have identified a paucity of coding point mutations relative to other cancer types, and none strongly correlated with grade. Germline genetics also play a key role: ∼57% of variability in prostate cancer diagnosis is explained by genetic factors (15). Polygenic risk scores (PRS) based on common germline variants can predict risk of a prostate cancer diagnosis (16) and may inform on disease aggression (17, 18). Rare germline variants in DNA damage repair genes or transcription factors like *HOXB13* are associated with increased risk of diagnosis and increased disease aggression (19–21). Localized prostate tumors arising in men who carry deleterious germline *BRCA2* mutations have a somatic mutational profile resembling metastatic castrate-resistant disease (22), whereas specific germline SNPs are associated with *PTEN* deletion (23) and somatic point mutations in the driver gene *SPOP* (24). Accumulating evidence suggests both germline and somatic genetics influence prostate cancer evolution.

**Figure 1.**
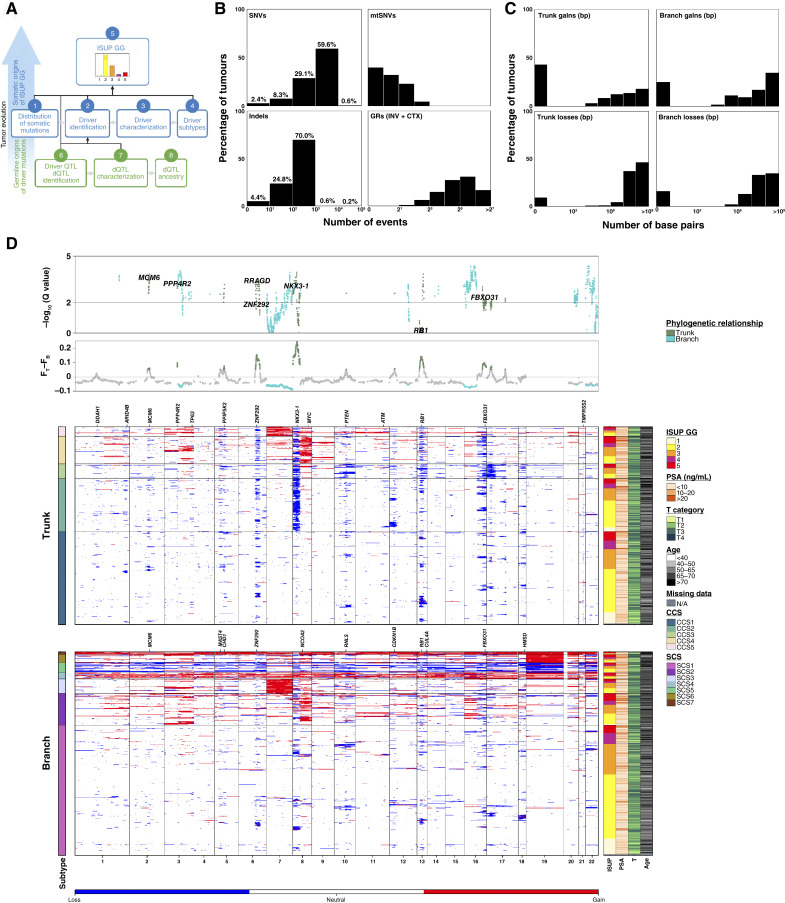
Mutation rates of prostate tumors. **A,** Schematic roadmap of the key analyses conducted in this study, offering insights into the genomic origins of the ISUP GG. **B,** Distributions of somatic mutation frequency for SNVs, indels, mtSNVs, and GRs across 666 tumor–reference WGS pairs. INV, inversions; CTX, interchromosomal translocations. **C,** Distributions of somatic clonal and subclonal CNA frequency (number of base-pairs) for losses and gains. **D,** Clonal and subclonal CNA landscape of localized prostate cancer. Heatmaps represent CNA profiles for the cohort split by CNA occurring clonally or subclonally. Columns represent genes and rows represent patients, grouped by subtype, then sorted by ISUP GG, and clustered within the GG. The top two panels show clonal–subclonal differences in CNA frequency (FTrunk – FBranch) of the dominant CNA type and statistical significance −log_10_(*Q* value) calculated using Pearson’s *χ*^2^ test with FDR adjustment. To avoid confounding by subclonal whole-genome duplication, patients with subclonal PGA >80% (9/664) were excluded from this statistical analysis. (**A,** Created with BioRender.com.)

To clarify the mutational and evolutionary drivers of prostate cancer grade, we studied 666 primary, localized prostate tumors with whole-genome sequencing (WGS; Fig. 1A). We created a compendium of 223 driver regions, most induced by structural variation undetectable by targeted sequencing. Many driver regions were altered by multiple mutation types; for example, *FOXA1* harbored point mutations in 5.8% of tumors but was mutated in other ways in an additional 10.1%. Using three-dimensional chromatin structure and enhancer profiling, we identified 35 germline SNPs that predict mutation of specific prostate cancer driver regions. Of these, 11 were validated in a 1,991-patient meta-analysis. Ten driver regions were more frequently mutated in high-grade cancers; these occurred early in tumor evolution and were associated with worse clinical outcomes. These data provide multiple lines of evidence supporting a model of germline and somatic genetics jointly driving the evolution of aggressive prostate cancer (Fig. 1A).

## Results

### Mutation Densities of Localized Prostate Cancer

We analyzed 666 localized treatment-naïve prostate tumors, each with WGS of the index lesion (61.0 ± 20.0 × depth, median ± SD) and WGS of matched reference tissue (36.0 ± 15.5×; Supplementary Fig. S1A), giving sensitivity to detect both clonal and subclonal variants. All samples were reviewed by a GU pathologist, and a region of the index lesion was selected for molecular analyses (Supplementary Table S1). These data comprised 251 newly sequenced and 415 previously sequenced tumor–reference WGS pairs (25–28). Detailed clinical information was collected, including pretreatment PSA abundance, age at diagnosis, clinical and pathologic T category, ISUP GG, and outcome measures. All sequencing data were analyzed through validated pipelines (Supplementary Fig. S1B) to identify nuclear and mitochondrial mutations (29–33).

As expected, prostate tumors harbored few somatic single-nucleotide variants (SNV), with a median 0.42 SNVs/Mbp sequenced in the nuclear genome, corresponding to ∼1,200 SNVs (Fig. 1B). About 14% of tumors were hypomutated (<0.1 SNVs/Mbp, ∼285 nuclear SNVs), whereas 0.6% were highly mutated (>5.0 SNVs/Mbp, ∼14,250 nuclear SNVs). One highly mutated tumor arose in a patient of African ancestry (34). The rate of nuclear somatic SNVs was well-correlated to that of insertions and deletions (indels; Spearman’s ρ = 0.75; Supplementary Fig. S1C), with a median 0.051 indels/Mbp. Most indels were short: 69.5% of one bp and 8.1% of two bp (Supplementary Fig. S1D). The median tumor harbored 26 nuclear coding somatic SNVs and indels and one mitochondrial SNV (Supplementary Fig. S1E). Extensive structural variation was common, with a median 32 genomic rearrangements (GR: inversions and interchromosomal translocations; Fig. 1B; Supplementary Fig. S1F). To quantify the evolutionary timing of CNAs, we applied a validated subclonal reconstruction strategy (35) to annotate each event in each patient as clonal (trunk) or subclonal (branch). A median 5.6% (trunk: 2.9%, branch: 1.7%) and 1.9% (trunk: 0.01%, branch: 0.5%) of the nuclear genome were deleted and gained, respectively (Fig. 1C).

Replicating prior observations in ISUP GG 2 and 3 tumors (36), the densities of almost all types of mutations were strongly correlated (Supplementary Fig. S1G). The more mutations a prostate tumor had of any single type, the more mutations it was likely to have of other types. These correlations do not reflect differences in tumor cellularity or ISUP GG and persist when considering only high-purity tumors of a single GG (Supplementary Fig. S1H). A subset of prostate tumors had very few somatic mutations of any type, consistent with previous studies (9, 13). This mutationally quiet subset may reflect epigenomic or transcriptomic dysregulation or substantial subclonal variation at levels undetectable by bulk sequencing and remains largely unexplained.

### The Evolutionary Paths of Prostate Cancer

Localized prostate cancer is strongly driven by copy-number changes, with specific events initiating malignant transformation and subsequent metastatic spread (9, 36). About a third of CNAs occurred before the most recent common ancestor (i.e., subclonal diversification) and appeared clonal, with a subset of tumors showing signs of subclonal whole-genome duplication [Fig. 1D (bottom) panel]. Because of tumor spatial heterogeneity and technical limits on subclonal detection (purity, ploidy, cancer cell fraction, and read depth), this is a lower bound on the subclonal fraction: most CNAs occur later during prostate tumor evolution.

We created subgroups using consensus techniques (37), resulting in five clonal copy-number subtypes (CCS; CCS1 through CCS5) and seven subclonal copy number subtypes (SCS; SCS1 through SCS7; Fig. 1D). These recapitulate and expand upon previous subtypes generated with lower-resolution methods and in smaller cohorts (7, 9, 13). Clonal and subclonal CNA features showed clear interrelationships (Supplementary Fig. S2A). Both clonal and subclonal CNA subtypes were tightly associated with ISUP GGs (Kruskal–Wallis test; *Q* < 0.01; Supplementary Fig. S2B).

We identified 31 driver regions of recurrent clonal and subclonal CNAs using GISTIC (Supplementary Fig. S2C; Supplementary Table S2). Most CNA drivers tended to occur early in cancer evolution: 21/31 were preferentially clonal. The remaining 10/31 occurred at indistinguishable frequencies between clonal and subclonal epochs, whereas no CNA drivers were preferentially subclonal [Fig. 1D (top) two panels]. This suggests that either most CNAs identified as subclonal do not provide a selective advantage to localized prostate tumors or there is very large heterogeneity in the selective pressures experienced by tumors during this evolutionary epoch. We used matching mRNA abundance data in 207 tumors to identify concordant CNA and mRNA changes and integrated these with literature reports to identify a putative driver gene for each recurrent CNA (see “Methods”; Supplementary Fig. S2D).

### Driver Regions of Localized Prostate Cancer

To supplement these 31 CNA-driver regions, we identified non-CNA mutational events subject to positive selective pressure during localized prostate tumor initiation and progression. We applied ActiveDriverWGS, which uses a generalized linear model to nominate recurrently mutated elements as candidate driver regions, adjusting for local mutational signatures (38). We identified 39 candidate driver regions affected by SNVs and indels: 13 protein-coding, 24 non-protein-coding, and two mitochondrial. The 24 non-protein-coding regions included 10 promoters, 10 enhancers, two long non-coding RNAs (lncRNA), one miRNA, and one 3’ untranslated region (UTR; Supplementary Fig. S3A). We applied ActiveDriverWGS to identify recurrent copy-neutral GRs, identifying 110 driver regions, including many well-characterized tumor suppressors (Supplementary Table S2). To produce an exhaustive compendium of driver regions in localized prostate cancer, we also included 58 low-frequency driver genes harboring protein-coding driver point mutations in a nonoverlapping cohort of 1,013 primary and metastatic prostate cancer exomes (14).

**Figure 2.**
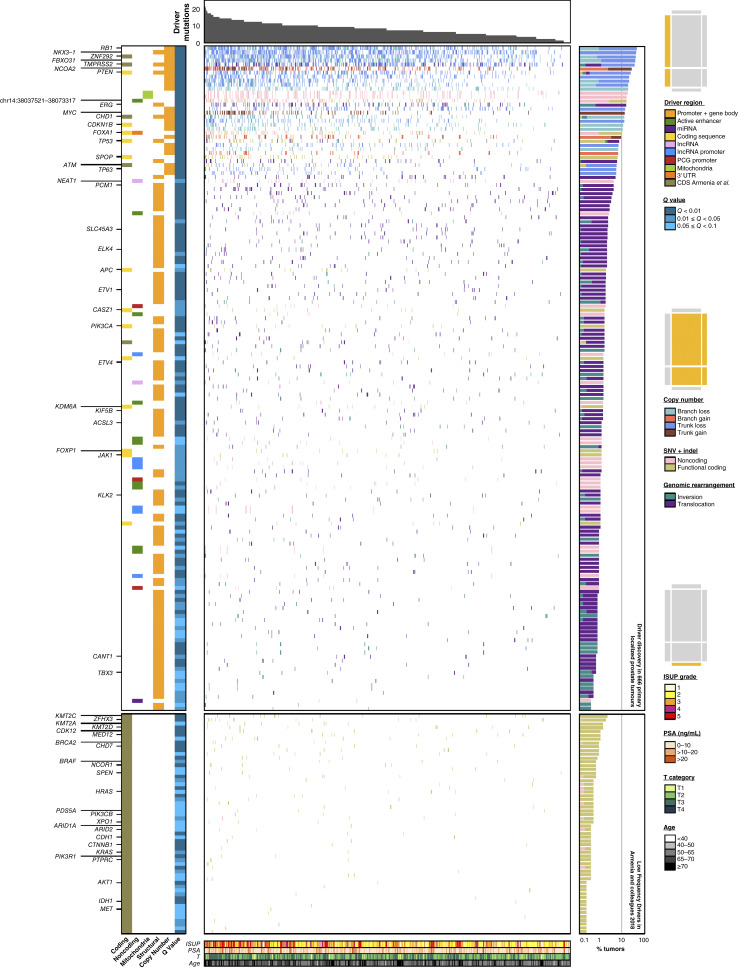
Somatic driver mutations in localized prostate cancer. Driver mutation discovery in 666 localized prostate tumors. The top barplot shows the distribution of the number of drivers in patients; the covariates on the left show the region type and statistical significance from ActiveDriverWGS and GISTIC. The top heatmap shows drivers found in this study (rows) for each patient (columns). The bottom heatmap shows drivers found in an exome meta-analysis (14). Heatmaps are colored by mutation type. Right barplot shows the number of drivers per patient. Bottom covariate bars show clinical features of patients. Gene labels on the left are for rows identified by tick-marks on the axis. Schematics above each set of legends indicate the panels to which they apply in yellow. For example, the top set of legends apply to the regional covariate heatmaps on the left side of the plot.

This final compendium of driver regions includes 31 CNAs, 110 GRs, and 97 SNVs/indels. In 20 cases, multiple types of mutations affected the same region, leading to a final compendium of 223 driver regions: 201 protein-coding genes and 22 non-coding regions (Fig. 2). The median tumor had eight somatic driver mutations. Only 2% of tumors harbored no driver mutations; these had lower pretreatment PSA (median 4.9 ng/mL *vs.* 7.6 ng/mL; Wilcoxon rank-sum test, *P* = 0.03). The number of driver regions mutated was not correlated with sequencing depth and tumor purity as sufficient coverage and tumor purity was achieved in each sample (Supplementary Fig. S3B). Almost all driver regions (97.3%) were more frequently altered by structural variation (CNAs and GRs) than by simple somatic mutations (SNVs and indels); *MED12* was a notable exception (Supplementary Fig. S3C). We verified the previously reported association between *TP53* mutations and genome instability (two-sided Wilcoxon rank-sum test, *P* = 4.01 × 10^−13^; Supplementary Fig. S3D; ref. 39).

Our compendium of 223 driver regions includes well-studied mutations like *ETS* gene fusions (51.8% of patients), SNVs in the mitochondrial control region (17.9%), and inactivation of *RB1* (43.2%) and *PTEN* (23.4%). Six driver regions previously described as harboring recurrent coding SNVs and indels, including *ATM*, *ZNF292*, and *STAB2*, overlapped with CNA and balanced GR driver regions. Several protein-coding genes were significantly affected by multiple mutation types, including key tumor suppressors like *TP53* and *PTEN*. Amongst these was the *FOXA1* locus, which has been reported to harbor coding point mutations in ∼3% of localized tumors (13), 3′ UTR indels in an estimated ∼9% of metastatic tumors (40), and frequent structural rearrangements (∼11 to ∼35%) across a spectrum of primary and advanced disease (41, 42). We identified a similar rate of non-synonymous SNVs (2.4%), along with recurrent coding indels (3.3%). These coding mutations were accompanied by a significant enrichment of SNVs and indels in the *FOXA1* 3′ UTR (5.2% of tumors; *Q* = 2.14 × 10^−25^). There was also a significant enrichment in non-coding SNVs and indels in an adjacent active enhancer region (chr14: 38037521−38073317, 4.8% of tumors; *Q* = 3.63 × 10^−19^), corroborated by H3K27Ac chromatin immunoprecipitation sequencing (ChIP-seq) in matched samples (43). Motif analysis predicted transcription factor–binding motif disruption in about half of patients with non-coding SNVs (44). These various types of mutations in the *FOXA1* locus were consistently associated with elevated *FOXA1* mRNA abundance (two-sided Wilcoxon rank-sum test, *P* = 6.06 × 10^−3^; Supplementary Fig. S3E). One *FOXA1* mutation occurred in 14.4% of patients, whereas an additional 1.5% carried two separate mutations, suggestive of biallelic inactivation. Tumors with wild-type *FOXA1* were prone to epigenetic dysregulation in four upstream cytosines that precede a guanine residue (CpGs) whose methylation was associated with *FOXA1* mRNA abundance (Supplementary Fig. S3F; ref. 45). These data reflect the multitude of ways that *FOXA1* can be dysregulated in primary disease, implicating a greater number of tumors than previously recognized by exome sequencing.

### Driver Region Mutations Shape Tumor Evolution

**Figure 3.**
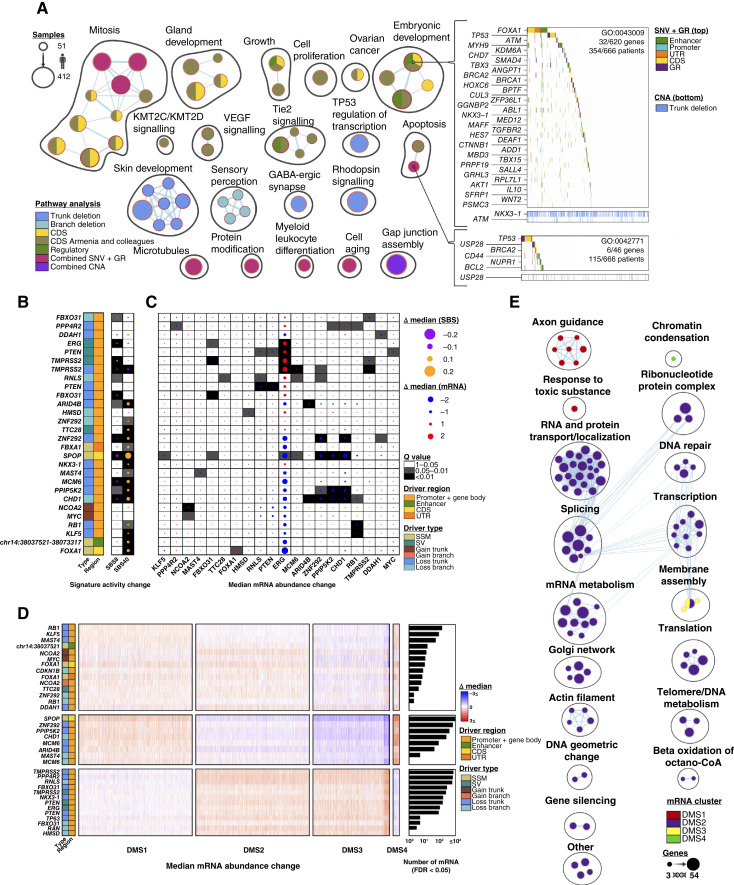
Functional characterization of driver mutations. **A,** Network diagrams represent multimodal pathway enrichment analysis of driver genes. Mutation types (i.e., the type of driver analysis) are indicated by shading of circles. Circle size represents the number of patients. Heatmaps show mutations in the cohort that affect genes contributing to two exemplar pathways dysregulated by multiple mutation types. The apoptosis pathway (GO:0042771 – intrinsic apoptotic signaling pathway in response to DNA damage by p53 class mediator), was identified to be significant only in the context of integrating statistical significance from all GR, and SNV, and indel driver analyses in coding and regulatory elements. The embryonic development pathway (GO:0043009 chordate embryonic development) was identified to be independently significant using SNVs and indels in regulatory elements, in coding elements, and in the Armenia and colleagues (14) dataset. Bottom covariates on the heatmaps show CNAs in pathway genes identified in driver CNA peaks (analyzed using GISTIC2). **B,** Associations between driver events and SBS signatures. Dot size and dot colors indicate median difference of signature activity. Background shading shows *Q* values from the Wilcoxon rank-sum test with FDR adjustment. Drivers and SBSs were ordered using hierarchical clustering. **C,** A summary of associations between driver events and mRNA abundance of driver genes. Dot size and colors indicate median difference of mRNA abundance. Background shading shows *Q* values from the Wilcoxon rank-sum test with FDR adjustment. **D,** Consensus clustering of 3,318 dysregulated mRNAs associated with driver mutations. Colors in the heatmap indicate median difference of mRNA abundance between patients with and without a specific driver mutation. Driver mutation type is on the left using colors from **A**. The right barplot shows the number of transcripts significantly associated with each driver mutation. **E,** Pathway enrichment analysis on the four mRNA subtypes from **D**. Clusters of biologically similar pathways are labeled and outlined for each subtype. The size of the pathway is indicative of the number of enriched genes. For (**B** and **C**) CNA drivers in patients with subclonal PGA >80% were excluded, and only driver events significantly associated with either SBS signatures or mRNA abundances are shown. (**A,** Created with BioRender.com.)

To determine whether our compendium of 223 driver regions reflects a smaller number of functional groups, we performed multimodal pathway analysis (46). Fifty pathways were subject to recurrent mutation, including apoptosis, mitosis, and embryonic development (*Q* < 0.05; Fig. 3A). Many pathways were altered by multiple mutation types. For example, both coding and non-coding driver SNVs and indels [i.e., either directly overlapping genes or distally associated through chromatin loops (38, 47)], preferentially affect growth and embryonic development pathways. Several pathways were recurrently altered by many low-frequency driver events (Supplementary Fig. S4A).

To determine whether driver events influenced downstream mutational processes, we quantified single-base substitution (SBS) signatures for each tumor (48). Common signatures included SBS8 [homologous recombination (HR) or nucleotide excision repair (NER) deficiency] and SBS40 (age-correlated), consistent with previous reports (49, 50). Of the 223 driver regions, 21 were associated with changes in mutational signature exposures (*Q* < 0.05; Fig. 3B, Supplementary Fig. S4B). Similarly, 18 driver events were associated with *cis* mRNA changes, and 34 with changes in *trans* affecting other driver genes (*Q* < 0.05; Fig. 3C). For example, *SPOP* mutant samples showed reduced *CHD1* mRNA abundance as expected (13).

These *trans*-associations between somatic mutations in one driver region and altered transcription of another led us to quantify the overall transcriptomic effect of driver events. A total of 3,318 transcripts were associated with one or more drivers. These defined four dysregulated mRNA subtypes (DMS; DMS1 through DMS4; *Q* < 0.05; Fig. 3D). For example, samples with *SPOP* and *ZNF292* mutations show similar patterns of transcriptomic dysregulations. Drivers that promoted the DMS1 transcriptional phenotype led to changed regulation of axon guidance, whereas those that promoted the DMS2 transcriptional phenotype dysregulated gene regulation very broadly (Fig. 3E). These data begin to systematically annotate our driver compendium with specific somatic molecular and evolutionary characteristics.

### Integrated Molecular Subtypes of Localized Prostate Cancer

**Figure 4.**
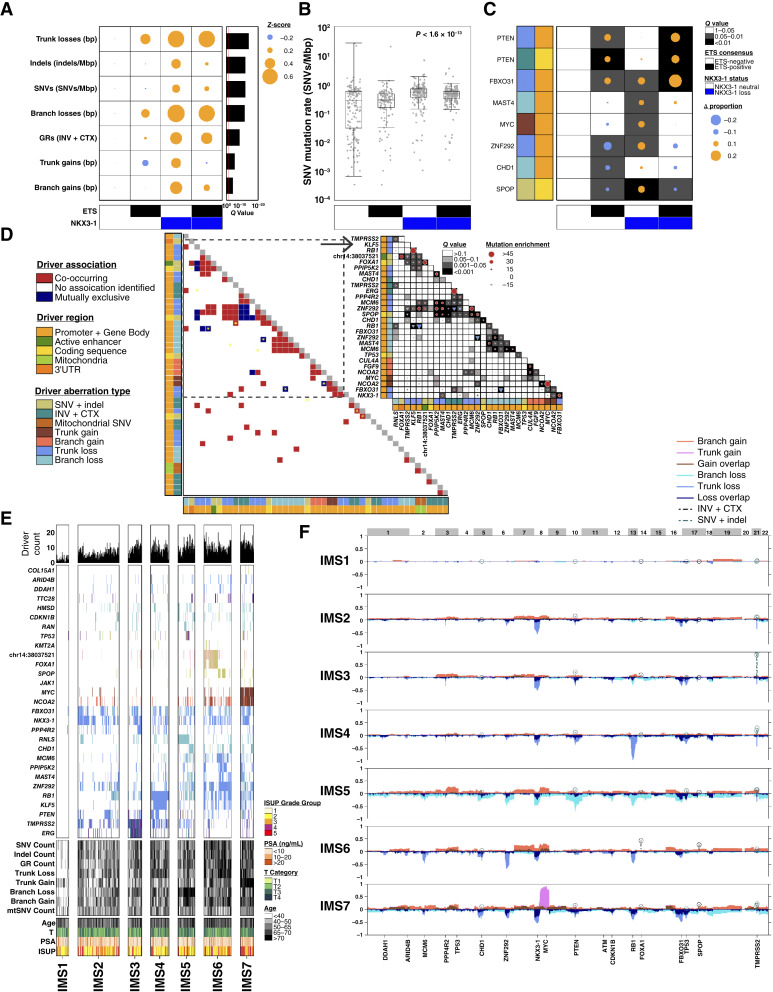
Mutational subtypes of localized prostate cancer. **A,** Mutation densities (rows) differ by ETS fusion and *NKX3-1* CNA status (columns). Dot size and color gives effect-size as a *Z*-score, scaled to ETS-negative, *NKX3-1*–neutral patients. The barplot on the right shows the FDR-adjusted *P* values from nonparametric Kruskal–Wallis tests. **B,** Comparison of log_10_-transformed SNV mutation rate for patients divided by ETS fusion and *NKX3-1* CNA status. *P* value is from a nonparametric Kruskal–Wallis test. **C,** Using a generalized linear model, eight driver mutations were identified whose frequency differed by ETS fusion and/or *NKX3-1* CNA status after FDR adjustment for multiple-testing. Dot size and color indicate the difference in proportion, scaled to patients with ETS-negative, *NKX3-1*–neutral tumors. Background grayscale represents *Q* values from a proportion test. **D,** Co-occurrence and associations of driver region pairs across 666 localized prostate tumors. For each pair of driver regions, a hypergeometric test was used to assess whether more mutations were detected than expected by chance alone (co-occurrence) or fewer (mutual exclusivity) after FDR adjustment for multiple-testing (*Q* < 0.05). The bottom-left heatmap shows all driver pairs; the dotmap on the top right provides effect sizes (dots) and *Q* values for a subset. Yellow stars on the heatmap mark drivers which are the same gene in clonal and subclonal CNAs. Dot size reflects the difference in driver events, quantified as the observed number minus the expected number. Color indicates deviation direction. The red circle signifies more driver events than expected by chance, whereas the upside-down blue triangle indicates fewer events than expected. **E,** Clustering of driver regions identifies seven patient subtypes: IMS1–IMS7. Columns are patients. The bottom set of rows shows clinical characteristics, the second set shows mutation densities, and the third shows driver mutations whose frequency differs between subtypes (proportion test; *Q <* 0.05). The top barplot gives the number of mutated driver regions for each patient. **F,** Summary subtype profiles showing the proportion of patients in the subtype with certain aberrations. In the positive direction, the proportion of clonal CNA gains, subclonal CNA gains, select GRs, and select SNVs. The lollipops show the proportion of patients for GRs and SNVs. In the negative direction, the proportion of patients in the subtype with clonal and subclonal CNA losses is shown. For each subfigure, CNAs in patients with subclonal PGA >80% were excluded. INV, inversions.

Interrogating the evolutionary impact of each driver event in isolation fails to capture the full complexity of the prostate tumor genome due to co-occurrence of driver events. For example, *ETS* fusions and *NKX3-1* loss are two of the most recurrent driver mutations. Both occur very early in tumor evolution and are typically clonal (28, 51). They co-occur in 23.5% of tumors. *NKX3-1* deletion was associated with increased burden of essentially all types of somatic mutation, whereas *ETS* fusions were associated with elevated copy-number loss (Fig. 4A). Tumors lacking mutations in either of these drivers showed remarkably increased and unexplained variability in SNV mutation rates (Fig. 4B; Kruskal–Wallis test; *P* < 1.61 × 10^−13^). Eight driver mutations were associated with *ETS* status, *NKX3-1* deletion or both, including *MYC* gain and *PTEN* loss (Fig. 4C).

To extend this result, we considered all possible pairs of co-occurring driver mutations. Of the 61 driver events present in at least 15 patients, 82 pairs co-occurred and 17 pairs were mutually exclusive (hypergeometric test; *Q* < 0.05; Fig. 4D; Supplementary Fig. S5A; Supplementary Table S3). One group of co-occurring drivers included *SPOP* SNVs and clonal loss of *ZNF292* and *MCM6*. Well-known associations between *CHD1*, *SPOP*, and their mutual exclusivity with *TMPRSS2*–*ERG* gene fusions were recapitulated (52, 53). Clonal CNA drivers mostly co-occur with other clonal CNA drivers, SNV/indel drivers (e.g., *FOXA1* and *SPOP*), and GR drivers (*TMPRSS2*–*ERG*). By contrast, clonal gain of *MYC* co-occurred with four subclonal drivers: *CUL4A* gain, *FGF9* gain, *ZNF292* loss, and *PPIP5K2* loss. These co-occurring and mutually exclusive driver pairs are insensitive to driver prevalence thresholds (Supplementary Fig. S5B; Supplementary Table S3).

Biallelic tumor suppressor inactivation was uncommon. Clonal and subclonal aberrations of CNA drivers for the same gene were frequently mutually exclusive, suggesting either selection against homozygous deletion or biases in modern CNA subclonal detection algorithms (Fig. 4D, marked with *). Similarly, there were no patients with both SNV/indels and CNAs/GRs for *PTEN* (Supplementary Fig. S5C), supporting the hypothesis that monoallelic *PTEN* losses are sufficient to accelerate tumorigenesis (54). We estimate that systematically evaluating mutual exclusive associations would require at least 1,063 tumors (*P* < 0.05) for a single tumor suppressor (Supplementary Fig. S5D). Thus, it is likely we have identified most co-occurring associations, but many mutually exclusive driver pairs remain unknown.

These data show context dependency in the effects of initiating mutations. Given the strong associations of both mutation density and specific drivers with one another and with clinical prognostic features, we sought to develop integrated genomic subtypes spanning all mutation types and grades of localized prostate cancer for the first time: all previous genomic subtypes have used only a subset of mutation types (9, 13, 55, 56). We generated seven integrated molecular subtypes (IMS; IMS1 through IMS7; Fig. 4E; Supplementary Fig. S5E). IMS1 is a mutationally quiet subtype comprising 9% of all patients (60/647). Tumors of this subtype have the lowest number of driver mutations (median: 2) and the lowest number of total mutations of all types and includes all 13 patients with no identified driver mutations (Supplementary Fig. S5F). Most IMS2 tumors carry *NKX3-1* deletions, with few other driver mutations (median: five drivers/tumor), in contrast to IMS3 through IMS7 which had more driver mutations (median: nine drivers/tumor). IMS3 tumors tended to show ETS fusions. Most IMS4 tumors had *RB1* loss. IMS5 tumors had increased subclonal copy-number losses. IMS6 tended to show loss of *ZNF292* and loss of *MCM6* and *SPOP* mutations. IMS7 tumors were characterized by gain of *MYC* (Fig. 4F; Supplementary Fig. S5G) and were of higher ISUP Grade (Pearson’s *Χ*^2^ test; *P* = 1.8 × 10^−5^). Subtypes were not biased by patient age, tumor extent, or pretreatment PSA but were associated with relapse after therapy (Supplementary Fig. S5H; *P* = 7.92 × 10^−6^; log-rank test), concordant with the clinical preeminence of grade in risk-stratification schemes.

### Molecular Correlates of Clinical Prognostic Features

**Figure 5.**
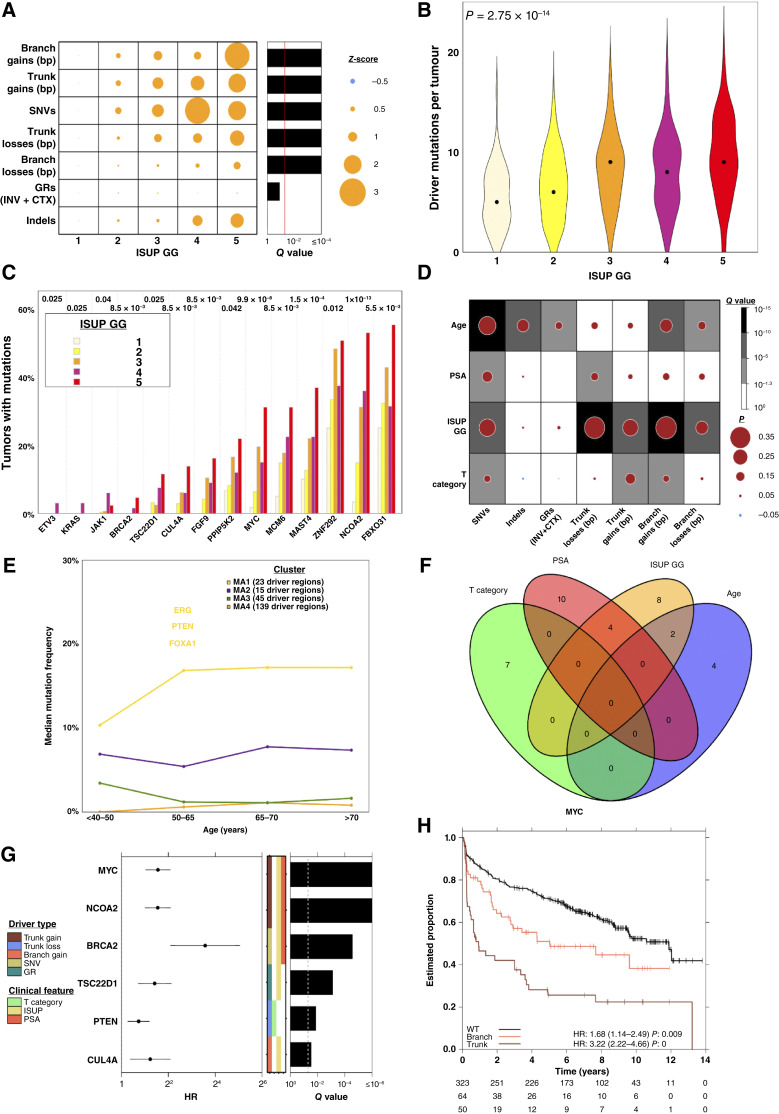
Mutational hallmarks of prostate cancer grade. **A,** A linear model was fit to relate each mutational density measure to ISUP GG using tumor and normal sequencing coverage as covariates. Dot size and color represents the effect size for each ISUP GG as a *Z*-score relative to ISUP GG 1. The barplot to the right shows the *Q* value from a nonparametric Kruskal–Wallis test. **B,** Distribution of the number of driver mutations per tumor in each ISUP GG; the median per GG is shown by a black dot. *P* value is from a one-way ANOVA. **C,** Genes whose mutation frequency is univariately associated with ISUP GG, ordered by the percentage of samples with mutations in ISUP GG 5 tumors. FDR-adjusted *P* values from the Pearson *χ*^2^ test are shown. **D,** Two-sided Spearman correlation between clinical covariates and measures of genomic instability with dot size showing the magnitude of correlation and background color representing the statistical significance. **E,** Consensus clustering identified four groups of genes with similar patterns of change across age categories. For each gene cluster, the median mutation frequency for each age category is shown, along with the number of genes in each cluster. **F,** Venn diagram of the driver genes that were statistically associated with clinical features. **G,** Cox proportional hazard models were fit for the driver regions that were associated with clinical features. Significant regions after FDR adjustment are shown, as well as the driver type and clinical feature the region was associated with. **H,***MYC* clonal and subclonal gains were associated with biochemical relapse. For each subfigure, CNAs in patients with subclonal PGA >80% were excluded. INV, inversions; WT, wild-type.

This strong association of mutational subtype with tumor grade led us to explore the mutational differences between tumors of different ISUP GGs. Controlling for tumor- and normal-sequencing coverage, higher-grade tumors were characterized by large increases in mutation burden (Fig. 5A). This was true for clonal and subclonal CNAs and for SNVs: there were a median 803 SNVs in GG 1 tumors *vs.* 1,401 in GG 5 (Supplementary Fig. S6A), recapitulating previous findings (9, 36). Despite our sample-size, we had limited power to detect grade associations for specific individual GRs and indels, reflecting a need for larger WGS cohorts (Supplementary Fig. S6B).

Higher-grade tumors had more driver mutations (one-way ANOVA; *P* = 2.75 × 10^−14^; Fig. 5B). Of the 223 driver regions, 14 were univariately associated with ISUP GG, most prominently *MYC* gain (Fig. 5C). Most grade-associated driver mutations preferentially occur clonally and early in tumor evolution: 9/14 driver regions associated with ISUP GG are CNAs, and 8/9 preferentially occur clonally. Drivers exhibited four broad types of association with grade (Supplementary Fig. S7A; Supplementary Table S3) which we termed driver clusters MG1 through MG4. MG1 was grade-invariant, whereas MG2 and MG3 showed a weak association with grade, consistent with rising genomic instability. MG4 comprised strongly grade-associated driver regions: a typical gene in MG4 was mutated in 28.3% of ISUP GG 1 but 49.5% of ISUP GG 5. There was also an association between ISUP GGs and IMSs (Pearson’s *Χ*^2^ test; *P* = 1.78 × 10^−5^; Supplementary Fig. S7B).

We extended these analyses to other clinical prognostic features of localized prostate tumors: age at diagnosis, pretreatment serum concentration of PSA, and tumor extent (T category). For each, we identified associations with both mutation density and specific driver mutations (Supplementary Fig. S7C–S7H). For example, age was associated with an increased burden of SNVs, indels and subclonal CNAs but intriguingly not of clonal CNAs (Fig. 5D and E). Six driver mutations were statistically associated with age at diagnosis, fourteen with serum PSA abundance, and seven with tumor extent (Supplementary Fig. S7F–S7H; Pearson’s *χ*^2^ test; *Q* < 0.1). Genes associated with different clinical features showed little overlap: no genes were associated with all four, consistent with their independent prognostic capacity (Fig. 5F). These data are consistent with disease of all grades emerging from a mutagenic field, with early clonal driver mutations informing the trajectory of low- versus high-grade.

We next identified genes associated with disease relapse after primary treatment (biochemical relapse, BCR), which is the main trigger for initiation of costly and morbid salvage therapies. We focused on the 35 genes associated with clinical prognostic features and mutated in at least 1% of patients (Supplementary Table S3). Six of these were associated with outcome (Fig. 5G), and five remained significant after adjustment for clinical prognostic features (Supplementary Fig. S7I; Supplementary Table S3). Of these five, *MYC* and *NCOA2* gain frequently co-occurred as both are on chromosome 8q (Fisher exact test; *P* = 2.16 × 10^−36^) whereas all other pairs did not (Supplementary Fig. S7J–S7K; Fisher exact test; *P* = 0.26).

Mutation timing was a major determinant of the impact of a mutation on patient outcome, with earlier mutations generally showing larger effects. Clonal *MYC* gain was a stronger prognostic feature than subclonal *MYC* gain (Fig. 5H; HR_clonal_ = 3.22 *vs.* HR_subclonal_ = 1.68). Similarly, clonal but not subclonal loss of *PTEN* was associated with worse outcome (Supplementary Fig. S7L). Thus, most driver mutations occur early in prostate cancer evolution and are likely associated with disease initiation. A small subset of these early-arising drivers are also associated with specific clinical prognostic features and progression to lethal disease.

### Germline Correlates of Somatic Mutational Drivers

Given that prostate cancer is one of the most heritable solid tumor types (15), we next sought to determine whether specific driver mutations were associated with specific germline SNPs. We term these relationships driver quantitative trait loci (dQTL). Focusing on 427 tumors derived from patients of European descent, we verified a lack of population substructure using identity-by-state clustering (Supplementary Fig. S8A; Supplementary Table S1) and restricted the analysis to 17 somatic drivers occurring in at least 5% of patients (range: 5.1%–57.3%) with robust literature support. These included 14 CNAs, two SNVs, and the fusion of *TMPRSS2* and *ERG* (T2E; Supplementary Fig. S8B; Supplementary Table S4). None of these drivers was associated with a PRS for prostate cancer incidence (Supplementary Fig. S8B; ref. 16).

As a positive control, we replicated previously reported SNP–driver associations. Two SNPs associated with T2E were replicated: rs16901979 (OR = 0.50; *P* = 3.90 × 10^−2^; Supplementary Fig. S8C) and rs1859962 (OR = 1.52; *P* = 5.05 × 10^−3^; Supplementary Fig. S8D; ref. 57). Two SNPs in *HSD3B1* previously associated with overall survival in advanced prostate cancer (58) were associated with tumor extent at diagnosis (Supplementary Fig. S8E and S8F) and showed trend associations with metastasis-free survival (Supplementary Fig. S8G and S8H). *APOE* SNPs associated with metastasis-free survival were validated (*P* = 0.027; Supplementary Fig. S8I; ref. 59). Tumors with the *APOE2* genotype had a significantly higher burden of GRs than *APOE4* tumors (OR = 0.45; *P* = 0.05; Supplementary Fig. S8J). We replicated previous reports of mutual exclusivity of a 3′ UTR germline variant in *TP53*, rs78378222, and *TP53* somatic alterations (Supplementary Fig. S8K). SNPs reported to be associated with *PTEN* loss (23) and *SPOP* point mutations were not replicated in this cohort (24). These positive controls confirm most prior germline–somatic associations but highlight the potential for false negatives in moderate cohort sizes.

**Figure 6.**
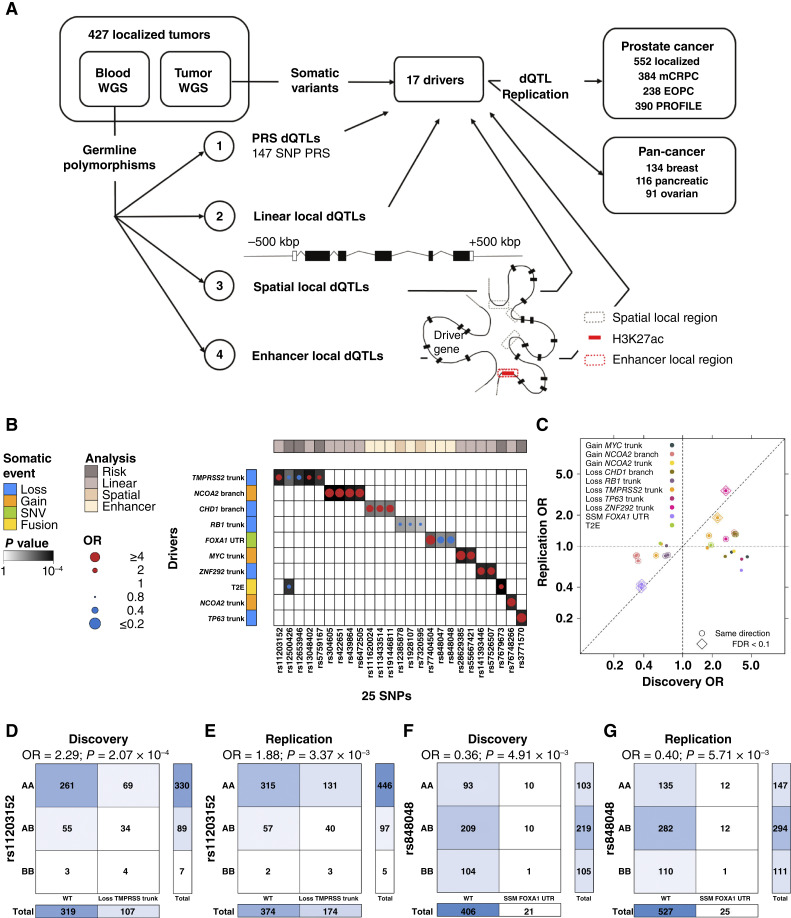
dQTLs bias somatic mutational landscape. **A,** Schematic of dQTL detection. The PRS used was by Schumacher and colleagues (16). Linear local dQTLs were assessed within ±500 kbp around a driver. Spatial local dQTLs were evaluated using regions defined by RNA Pol-II ChIA-PET profiling in LNCaP, DU145, VCaP, and RWPE-1 cell lines and RAD21 ChIA-PET in LNCaP and DU145 cells. Enhancer regions were defined using H3K27ac HiChIP profiling in LNCaP cells. All discovered dQTLs were tested for replication in six replication cohorts. **B,** Summary of 26 dQTLs involving 25 unique variants. Dot size and color indicate the magnitude and direction of ORs between the SNP and somatic driver. Background shading indicates *P* values. Covariate on left indicates type of somatic mutation; the top covariate indicates the analysis strategy for the discovery cohort . **C,** Comparison of ORs in the discovery *vs.* replication cohort for tag dQTLs. Horizontal and vertical dotted lines represent OR = 1, and the diagonal line represents y = x. Halo around points indicates replication of direction, diamond around points indicates *Q* < 0.1 in the replication cohort, and dot color indicates the somatic driver. **D** and **E,** Contingency tables for rs11203152 association with clonal loss of *TMPRSS2* in (**D**) discovery and (**E**) replication cohorts. **F** and **G,** Contingency tables of rs848048 associated with SNVs in *FOXA1* 3′ UTR in (**F**) discovery and (**G**) replication cohorts.

Fully powered genome-wide association studies (GWAS) require many thousands of patients with tumor WGS, not yet available, thus we leveraged four targeted strategies to enrich for dQTL candidates (Fig. 6A). First, we tested germline risk variants known to be associated with risk of prostate diagnosis. Second, we identified local dQTLs: regions in close proximity to each somatic driver based on linear DNA sequence. Third, we identified spatial local dQTLs, defined by three-dimensional DNA structure. Fourth, we identified prostate enhancer–associated dQTLs.

We first considered 147 risk alleles (16), focusing on the 134 with a minor allele frequency (MAF) > 0.05 (Fig. 6A). Of these, six were associated with one or more somatic drivers (logistic regression; *Q* < 0.1; light pink in Fig. 6B; Supplementary Fig. S9A; Supplementary Tables S5 and S6). All six dQTLs remained significant after adjusting for index event bias by ISUP GG, T category, and PSA (*P* < 7.8 × 10^−3^).

Second, we evaluated common SNPs (MAF >0.05) within ±500 kbp of the somatic event boundaries (Supplementary Fig. S9B). The 17 somatic drivers were each compared with 1,332 to 11,618 germline SNPs (median = 2,279, haplotype blocks = 80–1,379; median haplotype block size = 7 SNPs; Supplementary Fig. S9C). After controlling for population structure and somatic mutation burden, 20 local dQTLs were identified in 11 haplotype blocks, involving five drivers (logistic regression; Bonferroni α = 0.1; *P*_unadjusted_ < 3.7 × 10^−4^; OR > 1.8; Fig. 6B; Supplementary Table S6). We selected one SNP to represent each haplotype block based on the minimum *P* value and verified 11/11 CNA tag SNPs using independent CNA array data in matched patients (Supplementary Fig. S9D).

Third, we defined proximity to the somatic event based on DNA secondary structure (Fig. 6A). Spatial local dQTLs were defined based on RNA polymerase II (RNAPII) ChIA-PET (60) and RAD21 ChIA-PET (61) in prostate cell lines. We considered regions outside the linear local boundaries if they interacted with the event region in at least two cell lines. Each of the 17 somatic drivers was evaluated for associations with 7 to 101 SNPs in this step (median = 32; haplotype blocks = 2–16; median haplotype block size = 3 SNPs; Supplementary Fig. S9E). Two dQTLs associated with clonal (trunk) loss of *RB1* were discovered (logistic regression; Bonferroni α = 0.1; *P*_unadjusted_ < 2.35 × 10^−2^; OR > 1.47; Fig. 6B; Supplementary Table S6). Both were verified using array-based CNAs (Supplementary Fig. S9F).

Fourth, we considered proximity as defined by interacting enhancers identified via HiChIP H3K27ac profiling in prostate cancer cells (Fig. 6A). We identified anchor pairs in which one anchor was within the driver region and the other outside of it (see “Methods”). The 17 somatic drivers were evaluated for associations with 0 to 1,059 SNPs (median = 35; haplotype blocks = 0–81; median haplotype block size = 5 SNPs; Supplementary Fig. S9G). We identified 11 dQTLs involving seven haplotype blocks and three somatic drivers (logistic regression; Bonferroni α = 0.1; *P*_unadjusted_ < 1.27 × 10^−2^; OR > 1.50; Fig. 6B; Supplementary Table S6). We verified 3/4 candidate CNA dQTLs using array-based data (Supplementary Fig. S9H).

### dQTLs Replicate across Stages of Progression and Cancer Types

**Figure 7.**
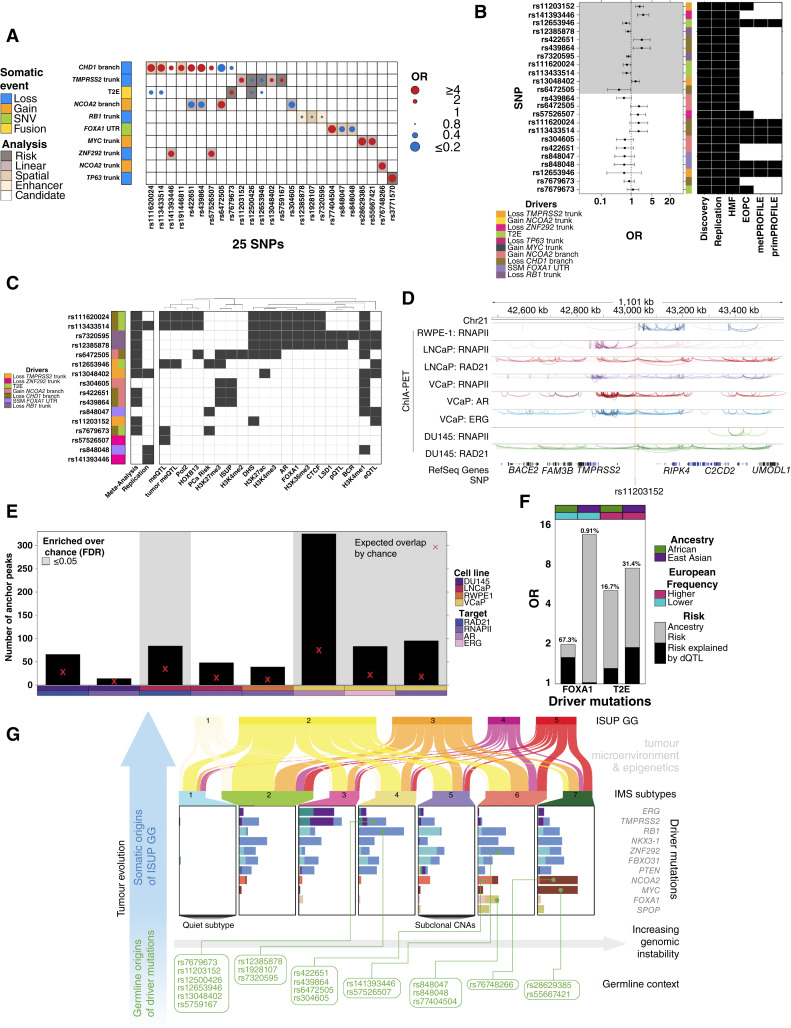
Characterization of dQTLs. **A,** Summary of all 35 dQTLs involving 25 unique SNPs. Dot size and color indicate the magnitude and direction of association (as OR), and background shading indicates dQTL discovered strategy. **B,** Forest plot of OR and 95% confidence interval for dQTL associations across 1,991 prostate tumors. Background shading indicates *Q* < 0.1. The middle covariate indicates the driver mutation, and the right heatmap indicates cohorts included in the analysis. **C,** Summary of molecular and clinical characterization of dQTLs. Gray indicates dQTL was association with methylation (meQTL), RNA abundance (eQTL), protein abundance (pQTL), transcription factor–binding, histone modification, ISUP GG, BCR, or risk of prostate cancer diagnosis (PCa Risk). Left covariate indicates somatic drivers. **D,** rs11203152 is located within regulatory dense region. Tracks show chromatin looping anchored by RNAPII, RAD21, AR, or ERG in RWPE-1, LNCaP, VCaP, or DU145 cell lines. **E,** The number of chromatin loops was higher than expected by chance in LNCaP and VCaP cell lines. Barplots shows the number of anchors within one Mbp of rs11203152. Bottom covariate indicates cell line and target, whereas background shading indicates significant enrichment (*Q* < 0.05). The red X indicates the expected number of chromatin loop anchors based on 100,000 randomly sampled, equally sized regions. **F,** dQTLs may explain differences in somatic mutation frequencies across ancestries. Barplot shows the risk of acquiring a *FOXA1* SNV or T2E in African (green) or Asian (purple) ancestry relative to European ancestry. The estimated percent of this risk explained by rs848048 (*FOXA1*) or rs11203152 (T2E) is indicated above the bar. The top covariate indicates ancestry: African in green and Asian in purple. Somatic mutation direction relative to European ancestry is indicated as higher (pink) *vs.* lower (teal). **G,** Schematic overview of primary prostate cancer evolution into ISUP GGs. The vertical blue arrow illustrates the temporal relationship between the germline context and driver mutations and the roles they play in tumor evolution. The germline SNPs (bottom) were found to be associated with driver acquisition illustrated by connecting lines. The somatic driver mutation frequency across IMSs is visualized using barplots. The horizontal arrow indicates increasing genomic instability across subtypes and ISUP GGs (9). The Sankey plot connects the IMSs and ISUP GGs, indicating the nondeterministic association between driver acquisition and clinical presentation, while noting the potential role of the tumor microenvironment (90) and epigenetics (45) (**G,** Created with BioRender.com.)

Our four dQTL discovery strategies identified 26 tag dQTLs involving 25 unique loci (Fig. 6B). Of these, 16 showed consistent effect sizes in a 552-patient replication cohort, mostly with exome-sequencing data (Fig. 6C). These included four very strong effects: rs11203152 with loss of *TMPRSS2* (a proxy for T2E status), rs141393446 with loss of *ZNF292*, and both rs848047 and rs848048 with SNVs in the 3′ UTR of *FOXA1* (Fig. 6D–G; Supplementary Fig. S10A–S10D). Focusing on the 16 dQTLs with consistent ORs in the replication cohort, we screened each tag SNP against all 17 somatic drivers in a candidate analysis. This identified nine candidate distal dQTLs (Fig. 7A; Supplementary Fig. S10E–S10G; Supplementary Table S7), of which seven replicated, for a total of 23 confirmed dQTLs.

To determine whether dQTLs affect multiple cancers, we tested the 20 tag dQTLs that influenced drivers mutated in ≥5% of Pan-Cancer Analysis of Whole Genomes (PCAWG) ovarian, breast, or pancreatic cancers, cancer types with sufficient sample size (*n* > 91), known heritability (31%–36%; refs. 15, 62), and shared driver events (33). Consistent effect sizes occurred for 14/20 dQTLs in other cancer types (Supplementary Fig. S10H–S10J). The association between rs76748266 and *NCOA2* gain replicated in pancreatic cancer (OR_pancreatic_ = 6.47; Q_pancreatic_ = 1.56 × 10^−2^; Supplementary Fig. S10K and S10L). The association of rs11203152 with *TMPRSS2* loss was nominally significant in ovarian cancer (OR_ovarian_ = 4.87; Q_ovarian_ = 0.11; Supplementary Fig. S10M). Thus, a subset of dQTLs affect multiple cancer types.

Finally, to generalize our results, we conducted a meta-analysis across 1,991 European descent prostate tumors, including our discovery and replication cohorts, 238 early onset prostate cancer (EOPC) tumors (63), 384 metastatic tumors (64), and 91 metastatic and 299 localized prostate tumors from the PROFILE cohort (65). Of the 23 dQTLs that showed concordant effects in the discovery and replication cohorts, 11 were replicated dQTLs (*Q* < 0.1; Fig. 7B; Supplementary Table S8). Thus, dQTLs can generalize across stages of prostate cancer and to other cancer types.

dQTL discovery requires matched blood and tumor tissue profiles. Despite using the largest whole-genome sequenced prostate cancer cohort available, the statistical power available is smaller than that of modern GWAS cohorts. The low frequency of most prostate cancer somatic drivers (∼5–20%) further reduces the power of our analysis. For common somatic drivers (5%–20% frequency), we have at best 80% power to detect an OR above 2.0 (Supplementary Fig. S11A–S11C). We nevertheless identified 35 dQTLs involving 11 somatic drivers and 27 SNPs (Fig. 7A). We extrapolate at least 314 additional dQTLs remain to be discovered with similar effect sizes to those identified in this study (see “Methods”). Subthreshold analysis akin to that from many early GWAS analyses supports the notion of a large landscape of unidentified prostate dQTLs (Supplementary Fig. S11D–S11I).

### Molecular and Clinical Correlates of dQTLs

To support the functional consequences of dQTLs, we quantified their impact on tumor gene expression (Supplementary Fig. S12A). Deregulation of tumor methylation is one mechanism by which the germline genome influences cancer risk (45, 66). We leveraged methylome data for 226 discovery cohort patients, 412 replication cohort patients, and 47 histologically nonmalignant prostate tissues. We identified and validated 110 methylation quantitative trait loci (meQTL) involving eight dQTLs (*Q* < 0.1 in both cohorts; Supplementary Fig. S12B and S12C; Supplementary Table S9). This was significantly more than expected by chance alone (*P* < 10^−4^; n_observed_ = 110; n_expected_ = 10; permutation test). Three SNPs were involved in tumor-specific meQTLs: they were associated with methylation changes in tumor but not normal prostate (|β_tumor_| > 0.12; Q_tumor_ < 8.50 × 10^−2^; |β_reference_| < 0.63; Q_reference_ > 0.12; ref. 45).

To explore whether dQTLs were associated with other epigenomic features, we studied histone modifications in primary prostate tumors for H3K27ac (*n* = 92 patients), H3K27me3 (*n* = 76), and H3K4me3 (*n* = 56) and androgen receptor (*AR*; *n* = 88) binding (Supplementary Fig. S12A; ref. 67). Of the 16 tag dQTLs, representing total unique variants, 10 overlapped active regulatory regions: six dQTLs overlapped H3K27ac sites (2–89 patients), of which five also overlapped H3K4me3 (1–47 patients) sites (Supplementary Fig. S12D; Supplementary Table S9). Five dQTLs overlapped H3K27me3, one of which overlapped H3K27ac sites in other patients, indicative of bivalent chromatin. We replicated these findings in a second cohort of 48 primary prostate cancer tumors profiled via ChIP-seq for H3K27ac, H3K4me2, H3K4me3, FOXA1, and HOXB13 (Supplementary Fig. S12A and S12E; Supplementary Table S9). Two of five dQTLs at H3K27ac modification sites demonstrated allelic imbalance specifically in tumor tissue and not in normal tissue, indicative of allele-specific regulation (Supplementary Fig. S12E). Of the 16 dQTL tag SNPs, 13 overlapped with active regulatory regions and master transcription factor–binding sites in five prostate cell lines (Supplementary Fig. S12F; Supplementary Table S9; refs. 68–81). Figure 7C summarizes all associations of dQTLs with DNA-binding proteins. Thus a subset of dQTLs modulate DNA–protein interactions, a determinant of local mutation rate (82).

To begin to elucidate a mechanism of dQTLs, we focused on rs11203152 because it is associated with loss of *TMPRSS2*, for which AR binding has been implicated (83), and was one of four dQTLs that we replicated (Fig. 6D and E). rs11203152 is in close proximity to multiple chromatin-looping sites anchored by RNAPII, RAD21, AR, and ERG (Fig. 7D; ref. 60). To quantify the enrichment of regulatory chromatin loops near rs11203152, we tested whether the number of anchors within 1 Mbp of rs11203152 was more than expected by chance (permutation test; *n* = 100,000 randomly selected size-matched regions). Anchors in RAD21, RNAPII, AR, and ERG were all enriched around rs11203152 (Fig. 7E), suggesting this germline SNP might interact with AR regulation to promote loss of *TMPRSS2*.

Only two dQTL tag SNPs functioned as expression quantitative trait loci (eQTL) for their associated somatic driver gene (Supplementary Fig. S13A): one for *RB1* mRNA abundance and one for *TMPRSS2* (*Q* < 0.1; Fig. 7C; Supplementary Fig. S13B–S13D). These RNA changes propagated through to protein abundance (Supplementary Fig. S13E–S13G). dQTLs did not generally influence proximal gene expression, defined as ±500 kbp (Supplementary Fig. S13H), with only one additional eQTL identified for rs12653946 – *IRX4*, validating a previous study (Supplementary Fig. S13I; ref. 84). Similarly, only two dQTLs influenced distal gene expression (>500 kbp from the SNP; Supplementary Fig. S13J and S13K).

Next, we interrogated whether any of the 23 dQTLs were associated with the seven IMSs. Four risk dQTLs (rs848048, rs848047, rs12385878, and rs7320595) were nominally associated with the IMSs (*P* < 0.1; *Χ*^2^ test); however, these associations did not survive multiple testing corrections despite *P* values that were smaller than expected by chance (Supplementary Fig. S13L–S13P). Similarly, 10 of 16 unique dQTL variants were nominally associated with at least one SBS signature (*P* < 0.10; Supplementary Fig. S13Q), although, once again these did not survive multiple hypothesis testing correction.

Finally, given that many somatic mutations, along with the IMSs, correlate with prostate cancer aggression (9, 28), we evaluated whether dQTLs might predict specific clinical features. One dQTL was associated with biochemical relapse (Supplementary Fig. S14A and S14B; Supplementary Table S9). Four dQTLs were associated with ISUP GG at diagnosis (Supplementary Fig. S14C–S14F). One dQTL was associated with the risk of prostate cancer diagnosis (OR = 1.02; *P* = 0.05; Fig. 7C; Supplementary Fig S14G). Overall, we discovered a positive, though nonsignificant, association between dQTL burden and biochemical relapse and ISUP GG (HR_BCR_ = 1.08; OR_ISUP_ = 1.07; *P* < 0.18), suggesting that dQTLs may be valuable candidates to further refine prognostic PRS (Supplementary Fig. S14H). Figure 7C and Supplementary Tables S9 and S10 summarize the broad epigenetic, transcriptional, and clinicoepidemiologic correlates of dQTLs.

### dQTL Allelic Frequencies Are Biased across Ancestry Populations

It has been well established that genetic ancestry is associated with specific features of the somatic landscape of prostate cancer (85–89), but it is unknown whether specific germline SNPs contribute to a significant proportion of these differences. We first demonstrated that regions harboring dQTLs were not themselves enriched in somatic SNVs (*P* > 0.15; Poisson generalized linear regression; Supplementary Fig. S14I). This was consistent in breast, ovarian, and pancreatic cancers as well (Supplementary Fig. S14J–S14L). By contrast, all dQTL tag SNPs had significantly different variant allele frequencies (VAF) between European and African or East Asian populations (*Q* < 0.01; Fisher exact test; Supplementary Fig. S14M and S14N). dQTL tag SNPs had similar VAFs within European populations, demonstrating that they are not driven by population stratification (Supplementary Fig. S14O).

We next focused on SNPs associated with two mutations with strong ancestry associations: T2E and *FOXA1* (85–89). The T2E gene fusion occurs less frequently in individuals of African and East Asian ancestries. The rs11203152 dQTL was associated with increased risk of loss of *TMPRSS2* in both discovery and replication cohorts (Fig. 6B and C). Concordant with these ancestry trends, the VAF for this SNP was significantly lower in both African and East Asian populations compared with European (VAF_African_ = 0.066; VAF_East__Asian_ < 0.001; VAF_European_ = 0.10; *Q* < 0.01). The association of rs11203152 with loss of *TMPRSS2* showed a similar effect in 115 men of African ancestry (Supplementary Fig. S14P).

*FOXA1* SNVs are more common in men of African ancestry than in men of European ancestry (88), whereas in men of East Asian ancestry, a SNV hotspot not found in other ancestries is common (87). The rs848048 dQTL tag SNP was negatively associated with the occurrence of SNVs in the 3′ UTR of *FOXA1* (Fig. 6B). Concordant with these ancestry differences, the tag SNP had a significantly lower VAF in African populations than in European or Asian ones (VAF_African_ = 0.23; VAF_European_ = 0.49; VAF_East Asian_ = 0.46; OR = 0.36; *Q* < 0.1), thus potentially explaining the higher burden of *FOXA1* SNVs in the absence of this protective germline SNP. We tested the association between rs848048 and SNVs in the *FOXA1* UTR in 115 African men. The allele distribution was substantially different in African individuals compared with European individuals, and the association did not replicate in the African cohort (OR_African_ = 0.96; P_African_ = 1.00; Supplementary Fig. S14Q), supportive of a germline role in ancestry-related somatic differences. We estimate that 16.7% to 31.4% of the ancestral differences in T2E and 0.9% to 67.3% of the ancestral differences in *FOXA1* can be explained by individual dQTLs (Fig. 7F).

## Discussion

Every tumor is different, with a life history shaped by its encounters with mutagens, selective microenvironmental pressures (90), and stochastic processes (91). This life history occurs in the context of the patient’s unique germline genome. Subtle differences in germline structure or function have decades to exert their small effects to influence tumor evolution. Similarly, the stochasticity of which driver mutations occur early in tumor development creates a context that shapes subsequent tumor evolution.

A comprehensive pan-cancer exome driver study identified 299 distinct protein-coding drivers across the entire natural history of prostate cancer (92). In localized disease alone, WGS identified 223 recurrently mutated driver regions. These represent all classes of somatic mutations and target both coding and regulatory regions. Our compendium contains almost all driver regions altered in at least 2% of localized prostate tumors (36). Most prostate cancer driver regions, and 37% of driver mutations observed in patient tumors, are silent to classic exome-sequencing or copy-number analyses. *FOXA1* provides a salient example: 5.8% of tumors harbor protein-coding defects, whereas 10.1% harbor other mutation types, with transcriptional consequences.

Building on previous analyses of germline–somatic interaction (93–98), these data begin to quantify how this landscape of somatic drivers is influenced by the germline context of an individual patient’s genome in primary prostate cancer. Individual dQTLs might influence acquisition of somatic mutations through a variety of mechanisms. For example, if a germline SNP modulates activity of an oncogene or tumor suppressor, cells that acquire a somatic aberration in the same oncogene or tumor suppressor may develop a stronger fitness advantage and experience clonal expansion. dQTLs could also affect the structural orientation of the local chromatin, influence the activity of master regulators, or influence the efficiency of local DNA damage repair. This variety of potential mechanisms supports the idea of polygenic models, in which many SNPs modestly influence somatic driver acquisition. Our data show that at least a subset of dQTLs act as meQTLs, eQTLs, and pQTLs. dQTL discovery provides a novel way to prioritize candidate susceptibility variants to refine PRSs predictive of not only risk of prostate cancer diagnosis but also the molecular profile of the resulting tumor.

Similarly, many somatic drivers influence downstream mutational signatures and gene expression. However, several lines of evidence in our data strongly suggest that high- and low-grade tumors are different points on a single evolutionary trajectory, rather than representing largely distinct evolutionary paths. First, overall density of all types of mutations increases with tumor grade. Second, the number of driver mutations increases with tumor grade. Third, high-grade tumors have all the mutational features of low-grade tumors. Fourth, specific mutations like *MYC* and *BRCA2* are significantly more frequent in higher-grade tumors, and these mutations are typically clonal. Clinical prognostic features like pretreatment serum PSA abundances and tumor extent show similar trends but affect different genes, consistent with their use as independent prognostic features. These data are consistent with subclonal reconstruction studies (28, 51) and support the hypothesis that prostate cancers of different grades emerge from a common evolutionary origin or field effect, with the divergence triggered by early mutations during this expansion (99) and the nature of those mutations influenced by both random chance and the patient’s germline genome. Alternatively, significant dysregulation by the aforementioned factors sets a clone within the mutagenic field toward a unique evolutionary trajectory, culminating in a specific grade of cancer. These two hypotheses are not mutually exclusive; both processes may occur simultaneously in the same mutagenic field (Fig. 7G).

Importantly, our data are consistent with, and partially directly explain, differences in somatic mutation rates across ancestries. They highlight the ongoing need for additional, easily accessible multi-ancestric cohorts of cancer genomes.

## Methods

### Patient Cohort

All patients underwent image-guided external beam radiotherapy (IGRT) or radical prostatectomy (RadP) for pathologically confirmed prostate cancer and were hormone-naïve at the time of treatment. In the IGRT cohort, a single transrectal ultrasound–guided biopsy was obtained prior to treatment. Fresh-frozen RadP specimens were obtained from the University Health Network Pathology BioBank, Genito-Urinary BioBank of the Centre Hospitalier Universitaire de Québec-Université Laval (CHUQ-UL), or the Australian Prostate Cancer Research Centres Biorepositories at Epworth Hospital and the Garvan Institute. Whole blood was collected at the time of written informed consent, consistent with local Research Ethics Board and International Cancer Genome Consortium (ICGC) guidelines. Previously collected tumor tissue was obtained based on Research Ethics Board-–approved study protocols (UHN 06-0822-CE, UHN 11-0024-CE, CHUQc-UL 2012-913:H12-03-192). To confirm ISUP GG and tumor cellularity, all tumor specimens were independently evaluated by expert GU pathologists (TvdK, BT, and AR) on scanned hematoxylin and eosin (H&E)-stained slides. Serum PSA measurements were taken at diagnosis and are reported in ng/mL. For IGRT patients, BCR was defined as a rise in PSA concentration of more than 2.0 ng/mL above the nadir (after radiotherapy, PSA levels drop and stabilize at the nadir). For RadP patients, BCR was defined as two consecutive post-RadP PSA measurements of more than 0.2 ng/mL (backdated to the date of the first increase). If a patient has successful salvage radiation therapy, this was not considered BCR. If PSA continues to rise after radiation therapy, BCR is backdated to first PSA >0.2. If a patient gets other salvage treatment (such as hormones or chemotherapy), this is considered BCR. Pathologic (RadP samples) and clinical (IGRT samples) T category was reported by NCCN criteria (www.nccn.org/professionals/physician_gls/pdf/prostate.pdf). All patients were N0M0 as an entry criterion.

### Sample Processing

Canadian samples were processed as previously described (28, 36, 100). Briefly, H&E-stained sections were marked by a GU pathologist to indicate areas of at least 70% tumor cellularity. Following manual macrodissection or punching of a core from this region, DNA was obtained via a phenol:chloroform extraction protocol. DNA was extracted from whole blood using ArchivePure DNA Blood Kit (5 PRIME, Inc.) at the Applied Molecular Profiling Laboratory at the Princess Margaret Cancer Center. DNA was quantified using Qubit 2.0 Fluorometer (Life Technologies) and assessed for purity using a Nanodrop ND-1000 spectrophotometer. For Australian samples, tumor regions confirmed by H&E from fresh-frozen cores in OCT were isolated using a scalpel and placed in 700 μL RLT Plus buffer for immediate homogenization (TissueRuptor, Qiagen). DNA and RNA were simultaneously extracted using Allprep Micro Kit (Qiagen), including on-column DNAse digestion of RNA. Genomic DNA was extracted from fresh-frozen whole blood using DNeasy Blood & Tissue Kit (Qiagen). DNA quantity was checked using Qubit dsDNA HS Assay Kit (Invitrogen), and DNA quality was assessed by gel electrophoresis (0.8% w/v agarose gel).

### WGS

#### Canadian Cohort: Ontario Institute for Cancer Research

For samples sequenced at the Ontario Institute for Cancer Research, the detailed protocols of library preparation and WGS have been described previously (28, 36). For a subset of blood samples sequenced at Illumina, the Illumina FastTrack Sequencing service was used. Sample preparation is described at: www.illumina.com/content/dam/illumina-marketing/documents/services/FastTrackServices_Methods_Tech_Note.pdf.

#### Canadian Cohort: The Center for Applied Genomics

For samples sequenced at The Center for Applied Genomics, 0.5 to 1.0 μg genomic DNA with OD260-280 between 1.8 and 2.0 were used for genomic library preparation and WGS. The Center for Applied Genomics quantified DNA samples using Qubit High Sensitivity Assay and checked sample purity using NanoDrop OD260/280 ratio. DNA (100 ng) was used as input for library preparation using Illumina TruSeq Nano DNA Library Prep Kit Set A (12 Set A index tubes, 24-sample library preparation kit, Cat. # FC-121–4001, Cat. # C-121–4002) following the manufacturer’s recommended protocol. In brief, DNA was fragmented to 350 bp on average using sonication on a Covaris LE220 instrument; fragmented DNA was end-repaired, A-tailed and indexed TruSeq Illumina adapters with overhang-T were ligated to the DNA fragments. Libraries were validated on an Agilent Bioanalyzer High Sensitivity DNA Kit chip (Cat. # 5067-4626) to check for size and absence of primer dimers, and quantified by qPCR using Kapa Library Quantification Illumina/ABI Prism Kit protocol (KAPA Biosystems, Cat. # 796020400 from Roche). Validated libraries were pooled in equimolar quantities and paired-end sequenced on an Illumina HiSeq X instrument following Illumina’s recommended protocol (Illumina HiSeq X Ten Reagent Kit v2.5, Cat. # FC-501–2501) to generate 150 bp paired-end reads. Sequencing runs were within Illumina specifications, which includes >68% passing filter reads, a minimum of 330 million paired-end reads per lane, and >75% of bases > Q30 at 2 × 150 bp.

#### Canadian Cohort: Baylor College of Medicine

For samples sequenced at Baylor College of Medicine (Houston, Texas), libraries of ∼350 bp mode insert size were prepared on Beckman robotic workstations (Biomek FX and FXp models) using TruSeq Nano DNA Sample Prep Kit. Briefly, DNA (200 ng) was sheared into fragments of approximately 200 to 600 bp using the Covaris E210 system (96-well format, Covaris, Inc.), followed by purification of the fragmented DNA using AMPure XP beads. This was followed by DNA end repair and a double size selection using different ratios of Sample Purification Beads provided in TruSeq Nano Kit. This DNA was next 3′-adenylated and ligated to Illumina multiplexing PE adapters followed by PCR amplification for eight cycles. A set of 12 index adapters provided in TruSeq Nano Kit that carry 8 bp barcodes (Cat. # D701-D712) were used for this purpose. Post-PCR Library products were purified using Sample Purification Beads to remove excess adapters and adapter dimer products. Agilent 2100 Bioanalyzer was used to estimate library sizes and to quantify library yields. Libraries were normalized and pooled (3–7 plex) at equimolar concentrations, with pool concentrations quantified by qPCR assay using KAPA Library Quantification Kit (SYBR FAST qPCR Master Mix) for loading on HiSeq X Ten instruments. WGS was performed using Reagent Kit v2.5 (Cat. # FC-501-2501), and libraries were loaded at 300 pmol/L concentration to generate 150 bp paired-end reads. Normal samples were sequenced to a coverage depth of 34 to 38×, and tumor samples to 57 to 64× depth. Insert sizes were a median of 360 bp and had a mode at 370 bp.

#### Australian Cohort

Samples were sequenced at Macrogen, Korea. DNA was extracted from tissue and blood and 0.5 to 1.0 μg used to prepare sequencing libraries using TruSeq Nano DNA Library Kit (Illumina). Samples were fragmented using the Covaris ME220 (Covaris). Libraries were quality checked for fragment size and library size distribution on an Agilent Technologies 2100 Bioanalyzer (Agilent Technologies). The concentration of each library was then normalized and pooled. To ensure optimum cluster densities across every lane of every flow cell, prepared libraries were quantified using qPCR according to the Illumina qPCR Quantification Protocol Guide. Roche’s rapid library standard quantification solution and calculator was used to confirm library concentrations. Sequencing was performed using the Illumina HiSeq X Ten platform (Illumina) generating paired-end reads of 150 bases in length.

### WGS Variant Detection

#### Alignment

Each lane of raw sequencing reads was aligned against human genome reference build hs37d5 using Burrows–Wheeler Aligner (v0.7.12–0.7.15; ref. 101). Lane-level BAMs from the same library were merged, marking duplicates using Picard (v1.121–2.8.2; Supplementary Table S1; http://broadinstitute.github.io/picard). Library-level BAMs from each sample were merged without marking duplicates. In addition, for the Canadian samples, the Genome Analysis Toolkit (GATK v3.4.0–3.7.0; ref. 102) was used for local realignment and base quality recalibration, processing tumor/normal pairs together. Separate tumor- and normal-sample BAMs were generated, and their headers were corrected using SAMtools (v0.1.19–1.5; ref. 103). Sequencing coverage was computed using Picard (v.2.17.11) CollectRawWgsMetrics with default cut-off (Supplementary Table S1).

#### Germline Variant Detection

Germline SNPs and indels were identified using the GATK (v3.4.0–3.7.0). First, HaplotypeCaller was run on the normal and tumor BAMs together, followed by VariantRecalibrator and ApplyRecalibration. In addition, somatic variants and ambiguous variants that have more than one alternate base were removed. We referred to the GATK best practices to develop this pipeline (https://www.broadinstitute.org/gatk/guide/best-practices). Germline variants were used to filter somatic SNVs and indels.

#### Cross-Individual Contamination Check

To estimate cross-individual contamination level, GATK ContEst (v3.4.0–3.7.0; ref. 104) was applied to all normal and tumor sequences. Both sample and lane-level analyses were performed (Supplementary Table S11). Genotype information was obtained from the germline SNPs generated by the GATK (v3.4.0–3.7.0). Population allele frequencies for each SNP in HapMap (hg19) were downloaded from https://www.broadinstitute.org/cancer/cga/contest_download.

#### Callable Base Generation

Positions in mapped reads were deemed “callable” if covered ≥10× in normal and ≥17× in tumor, calculated using bedtools (v2.26.0; ref. 105), as previously described (36).

#### Germline Similarity Prediction

Germline similarity was predicted by computing distance matrices between each sample and the 1,000 genome reference set, including multiple populations using germline variants in the exome region (106). Then, the closest super population was assigned to the sample (Supplementary Table S1).

#### Nuclear Somatic SNV Detection

The pipeline to inform somatic SNV calling was designed based on the benchmarking results from Ewing and colleagues (29). Somatic SNVs were predicted using SomaticSniper (v1.0.5; ref. 107). First, somatic SNV candidates were detected using bam-somaticsniper with the default parameters except -q option (mapping quality threshold), which was set to one. To filter the candidate SNVs, a pileup indel file was generated for both normal and tumor BAM files using SAMtools (v0.1.6). SomaticSniper (v1.0.5) package provides a series of scripts to filter out possible false positives. First, standard and LOH filtering were performed using the pileup indel files. Then, bam-readcount (v0.8.0 dea4199) was run with a mapping quality filter -q 1 (otherwise default settings) in order to apply the false positive filter. Lastly, the high-confidence filter was applied with default parameters. The final VCF containing high-confidence somatic SNVs was used for downstream analyses.

#### Nuclear Somatic SNV Filtering

After somatic SNV calling using SomaticSniper, identified SNVs in positions that were not considered “callable” were removed and then passed through an annotation pipeline. SNVs were functionally annotated by ANNOVAR (v2017-07–16; ref. 108), using the RefGene database, with nonsynonymous, stop-loss, stop-gain, and splice-site SNVs considered functional. If more than one mutation was found in a sample for a gene, the mutation of the higher priority functional class was used for visualization. SNVs were filtered using the Perl library Bio::DB::HTS::Tabix (v2.10), removing SNVs found in any of the following databases: gnomAD (v2.0.2, variants with “PASS” flag; ref. 109), dbSNP (build 150, modified to remove somatic and clinical variants with the following flags: variant allele origin (SAO) = 2/3, variant is precious (PM), variation is interrogated in a clinical diagnostic assary (CDA), provisional third party annotation (TPA), is mutation (MUT) and has OMIM/OMIA (OM); ref. 110), 1000 Genomes Project (v3; ref. 111), Complete Genomics 69 whole genomes (112), duplicate gene database (v68; ref. 113), ENCODE DAC and Duke Mappability Consensus Excludable databases (comprising poorly mapping reads, repeat regions, and mitochondrial and ribosomal DNA; ref. 114), invalidated somatic SNVs from 68 human colorectal cancer exomes (unpublished data) using the AccuSNP platform (Roche NimbleGen), germline SNPs and indels from all samples in this study, and the Fuentes database of likely false positive variants (115). SNVs were whitelisted (and retained, independently of other filters) if they were contained within the Catalogue of Somatic Mutations in Cancer (COSMIC) database (v83; ref. 116). COSMIC variants detected only in publicly available samples used in this study were removed to avoid whitelisting variants using the samples they were discovered in. Furthermore, those variants flagged as SNPs, as “variants of unknown origin,” with FATHMM score <0.7, with FATHMM noncoding score <0.7, or identified in fewer than 10 samples were removed from whitelists. The mutation rate (SNVs/Mbp) was calculated by dividing the number of somatic SNVs after filtering by the count of callable loci (Supplementary Table S1).

#### Nuclear Somatic Indel Detection and Filtering

Small indels were called with cgpPindel v2.2.4 (117) with default parameters and the following genomic rules (F004, F005, F006, F010, F012, F018, F015, and F016) and soft rules (F017). Normal filtering was done using a panel of noncancer reference samples from the 1000 Genomes Project (pindel_np.gff3.gz). Filtering for simple repeats (simpleRepeats.bed.gz) and removal of bad anchors (ucscHiDepth_0.01_mrg1000_no_exon_coreChrs.bed.gz) was performed using data downloaded from the UCSC browser. Indels were also filtered using the Perl library Bio::DB::HTS::Tabix (v2.10), removing indels found in any of the following databases: gnomAD (variants with “PASS” flag; ref. 109), dbSNP (build 150, modified to remove somatic and clinical variants with the following flags: variant allele origin (SAO) = 2/3, variant is precious (PM), variation is interrogated in a clinical diagnostic assay (CDA), provisional third party annotation (TPA), is mutation (MUT), and has OMIM/OMIA (OM); ref. 110), 1000 Genomes Project (v3; ref. 111), ENCODE DAC, and Duke Mappability Consensus Excludable databases (114). Indels were whitelisted using the COSMIC database (v83) as described for somatic SNV. Gene annotation (human.GRCh37.indelCoding.bed.gz) was also provided. The resulting VCF files were annotated with SnpEff (v4.3R), and their functional categories were used downstream.

#### Nuclear Somatic Copy Number Aberrations

The pipeline to determine clonality of somatic copy number aberrations (CNA) was designed based on the results from Liu and colleagues (32). CNAs were identified using Battenberg (cgpBattenberg v3.3.0, Battenberg R-core v2.2.8, alleleCount v4.0.1, PCAP-core v4.3.2, cgpVcf v2.2.1, impute2 v2.3.3; ref. 118). Clonal and subclonal CNAs were predicted using the default cut-off of *P* value 0.05, which indicates whether the segment should be represented by clonal or sublconal state. Segments with *P* value < 0.05 were predicted subclonal. In addition, segments of length below 10 kbp were filtered out. The Proportion of the Genome with a Copy Number Aberration (PGA) was calculated as follows: (clonal PGA + subclonal PGA) ÷ genome length (3,137,161,264 bp). CNAs in samples with subclonal PGA >80% (indicative of a subclonal whole-genome duplication event) were excluded in downstream analysis, estimations of descriptive statistics, and data visualizations where indicated.

#### Nuclear Somatic GR Detection

The pipeline to identify somatic GRs was designed based on the benchmarking results from Lee and colleagues (31). Somatic inversions and interchromosomal translocations were predicted using Delly (v0.7.7–0.7.8; ref. 119) at a minimum median mapping quality of 20 and a paired-end and split-read evidence threshold of five reads. A list of somatic inversions and interchromosomal translocations were produced by removing germline GR mutations from the resulting VCF files, which were further filtered using a consolidated list of structural variants from the 666 blood reference samples of this cohort. To identify genes affected by the GRs, BED files were generated for each sample from deleted regions, and breakpoints from inversions, interchromosomal translocations, and tandem duplications. These BED files were annotated using SnpEff (v4.3R; ref. 120), and gene symbols extracted for downstream analyses.

#### Mitochondrial Somatic SNV Detection

Mitochondrial data analyses were performed largely as described previously (30). Briefly, for somatic mitochondrial SNV (mtSNV) calling, reads mapped to the mitochondria during whole-genome alignment were extracted using BAMQL (v1.4; ref. 121) as follows:

bamql -b -o out_mito_reads.bam -f input_wgs.bam “[chr(M) & mate_chr(M)] | [chr(Y) & after(59000000) & mate_chr(M)]”

BAMQL output files were realigned using MToolBox (v1.0; ref. 122) with default settings and parameters except gmap (v2017-10-12) and with the default RSRS as the reference genome (123). mitoCaller (v1.0; ref. 124) was used to obtain allele counts (base quality threshold >20) of each mitochondrial genomic position on realigned BAMs. The predicted mitochondrial genome for each tumor sample and the number of reads supporting each base were compared with the corresponding normal sample. Positions in which the absolute difference in heteroplasmy fraction (∆HF) was >0.2 and read depths >100 (both normal and tumor) were considered mtSNVs. HF estimates were adjusted to account for tumor cellularity using tumor purity (rho) value computed by ascatNGS (125).

### CNA Subtypes

Consensus clustering was applied separately to the clonal and subclonal CNA profiles using the ConsensusClusterPlus package (v1.40.0) with the following customized arguments (reps = 1,000, pItem = 0.8, distance = Jaccard, and clusterAlg = Ward.D2). The patients were further sorted by ISUP GG and then clustered again using Jaccard distance metric and hierarchical clustering (Ward.D2) for each ISUP GG. We then compared the presence of clonal and subclonal CNAs across the patients and identified significantly different genes. Genes that showed delta frequency more than 0.05 or less than −0.05 were tested using the Pearson’s *χ*^2^ test, and *P* values were FDR-adjusted. To test whether the CNA driver regions were altered at different frequencies clonally and subclonally, a logistic regression model was fit, using PGA as a covariate. The Kruskal–Wallis test was used to test whether the CNA subtypes were associated with each clinical feature (ISUP GG, PSA, T category, and age). *P* values from the Kruskal–Wallis tests were adjusted using the FDR. The Epsilon-squared (ε^2^) value between each clinical feature and CNA subtype was calculated for clonal and subclonal CNA subtypes.

### Driver Gene Identification

#### Nuclear CNAs

GISTIC (v2.0.23; ref. 126) was run on the cohort’s filtered copy number segments to identify focal driver CNAs. Chromosome Y’s copy number was not included in the analysis given the predominant presence of sequence assembly gaps. Parameters were set as follows: qv_thresh = 0.01, join_segment_size = 700, res = 0.01, and conf_level = 0.9. To determine whether there were transcriptomic changes between samples with a CNA compared with those without, a Wilcoxon rank-sum test was used using previously published RNA sequencing (RNA-seq) and mRNA microarray data (127, 128). When comparing samples with a copy-number loss, the alternative was set to less, and when comparing samples with a copy-number amplification, it was set to greater. *P* values were adjusted for multiple testing with the Bonferroni method.

#### ActiveDriverWGS

ActiveDriverWGS (v1.0.1) was used to identify driver mutations in protein-coding genes and associated noncoding regulatory elements (38). Briefly, the tool performs statistical analysis of the number of SNVs and indels within a region of interest (ROI) relative to the expected number of mutations in an adjacent background window using Poisson generalized linear regression. The expected number of mutations is adjusted for the length and sequence of the element and background based on the trinucleotide context of observed SNVs. Modifications to the tool were made for driver discovery on the mitochondrial SNVs and nuclear GR datasets, as outlined in the “Nuclear GRs” section. *P* values were adjusted for multiple hypothesis testing using the FDR on each set of elements.

#### Nuclear SNVs and Indels

Driver discovery on the somatic nuclear SNV and indel dataset was conducted using ActiveDriverWGS using a background sequence of 50 kbp. Input regions for driver discovery were adapted from the PCAWG consensus dataset for GRCh37, including coding sequences, promoters, 5′ UTRs and 3′ UTRs, enhancers, and lncRNAs (129). In addition, lncRNA promoters, defined as +2,000 bp upstream of the transcription start site (TSS), and miRNAs (from miRBase) were also included. Cis-regulatory modules were also defined by regions captured by ENCODE ChIP-seq datasets. Regions were included and merged if they were overlapping in at least two ENCODE cell lines, as previously described (38). Finally, enhancers potentially specific to prostate tumors were identified from H3K27Ac ChIP-seq experiments on a subset of the Canadian samples (43). Regions overlapping in more than half of samples (12 of 24) were included and merged.

#### Nuclear GRs

ActiveDriverWGS was used to find recurrent somatic GRs using inversion and translocation breakpoints. Because the recurrence of breakpoints is not known to be strongly dependent on local sequence context, they were considered equally likely to occur at any nucleotide in the ROI. A one Mbp background window was used to estimate breakpoint probability as GR recurrence is correlated with macrogenomic features such as GC content, replication time, and chromatin compartment (25). Due to the uncertainty of how GRs affect regulatory elements and the large number of lncRNAs that overlap protein-coding genes, only protein-coding genes, introns, and their directly associated regulatory elements (UTRs and promoters) were used to define ROI. These regions were merged for each gene.

#### Mitochondrial SNVs

Mitochondrial SNVs were filtered by a HF of 20%. Input regions were adapted from www.mitomap.org as detailed (30). Due to the large number of overlapping regions, only the regions with clear functional annotations were considered in addition to protein-coding and RNA genes: MT-CR (control region), MT-OL (light strand origin), MT-TER (transcription terminator), and MT-ATT (membrane attachment site). The background region used was the entire mitochondrial genome excluding the ROI.

#### ETS Consensus

Events involving *ERG* and *ETV* are collectively referred to as ETS events when *ERG*, *ETV1*, *ETV4*, *ELK4*, *TMPRSS2* or *SLC45A3* were detected by ActiveDriverWGS or GISTIC. ETS calls for Canadian samples were further augmented using ERG IHC, deletion calls between *TMPRSS2* and *ERG* loci in either array-CGH or OncoScan SNP array data, and transcript fusion calls in RNA-seq. In addition, ETS status from several published datasets (9, 13, 25, 27, 55, 56) were retrieved from Supplementary Tables or equivalent and merged with predictions from this study.

#### FOXA1

The mRNA abundance between samples with mutations in the exons, 3′ UTR, and the active enhancer (chr14 38037521–38073317) and samples without mutations were compared with a two-sided Wilcoxon rank-sum test. Noncoding mutations were analyzed for transcription factor–binding motif disruptions using FunSeq2 with default parameters (44). Spearman’s correlation was used to determine whether a correlation existed between the *z*-score of methylation and mRNA abundance in samples without mutations in the *FOXA1* locus (45).

### Pathway Analysis of Drivers

Pathway analysis was conducted using ActivePathways (v1.0.1; ref. 46). ActivePathways detects significantly enriched pathways by integrative analysis of multiple datasets. It prioritizes genes that are statistically supported with multiple types of evidence by applying a data-fusion procedure. Subsequently enrichment analysis of pathways and Gene Ontology terms is performed on the ranked and leniently filtered gene list. Default settings were used in this study: *P* value merging was conducted using Brown’s method of combining *P* values by accounting for *P* value dependencies. Multiple hypothesis testing correction of derived pathways was performed using the Holm–Bonferroni method. Ranked input genes were filtered using a cut-off of *P* < 0.1 prior to pathway enrichment analysis. Gene sets from the molecular pathways of the Reactome database (release 2018-06-01) and Gene Ontology database biological processes (annotations: Ensembl, classes 2019-06-05) downloaded from the g:Profiler website were used for pathway enrichment analyses (130). Gene sets containing fewer than three or more than 1,000 genes were excluded from analyses. Enriched pathways were filtered by a statistical threshold of 0.05 after Holm–Bonferroni adjustment. The *P* values of genes and regulatory regions calculated by ActiveDriverWGS for SNVs, indels, and GRs, the FDR-corrected *P* values of drivers reported by Armenia and colleagues (14) and the FDR-corrected *P* values calculated by GISTIC for CNAs were used as input to ActivePathways. CNAs were analyzed separately from other mutation types as they represent a region-based analysis rather than a gene-based analysis. Clonal and subclonal gains and losses were used as the input. Genes located within a recurrently amplified or deleted peak were associated with the FDR-corrected *P* value for that peak. For CNA analysis, pathways enriched for genes in only one or two peaks were discarded to prevent false positives resulting from families of adjacent genes (typically arising from gene duplication events). These included taste 2 receptors, keratin-associated protein genes, and the TNF receptor superfamily which enriched for pathways associated with keratinization and TRAIL binding. For SNVs and indels, up to four *P* values were used for each gene: coding sequences, promoters, UTRs, and noncoding regulatory elements. Noncoding regulatory elements included regions defined by H3K27Ac ChIP-seq on 24 tumors from this cohort (43), by the PCAWG enhancer dataset, and by the ENCODE cis-regulatory modules. Noncoding regulatory elements were associated with genes either by direct overlap of elements with promoters and transcribed sequences or through chromatin loops identified from HiC data (47). As there were two UTRs and possibly multiple noncoding regulatory elements for each gene, the region with the most significant *P* value was chosen for each. For GRs, the *P* value from ActiveDriverWGS conducted on breakpoint analysis for each gene was used.

### Association of Driver Mutations with Mutational Signatures

Signature Analyzer (131) was used to predict mutational signatures from the filtered somatic SNVs (see “Nuclear Somatic SNV Filtering”). Signature Analyzer was performed with 1,000 iterations, and COSMIC v3 was used as reference. Signatures with mean activity less than 5% were excluded from the analysis. Patients were divided into two groups based on the existence of driver mutations that affected at least 5% of the cohort. Then, for each driver, each of the median signature activity and an FDR-adjusted *P* value from a two-sided Wilcoxon rank-sum test between the two groups was computed.

### Association of Driver Mutations with mRNA Abundance

mRNA abundance data for 20,846 genes in 207 patients was used to investigate associations between driver events and global mRNA abundance. Patients were divided into two groups based on the existence of a driver mutation. For each driver, identified in at least 5% of the cohort, a two-sided Wilcoxon rank-sum test between the two groups was computed. *P* values were adjusted using the FDR method. Consensus clustering was applied to driver mutations and 3,318 mRNAs (*Q* value < 0.05 at least for one driver mutation) using the ConsensusClusterPlus package (v1.50.0) with the following customized arguments (reps = 1,000, pItem = 0.8, distance = Jaccard, and clusterAlg = Ward.D2). Three driver subtypes and four DMSs were identified.

### Pathway Enrichment Analysis for Dysregulated mRNA Clusters

Gene sets of interest for each DMS were processed using the g:Profiler2 R package (0.1.9; ref. 132) The list of all genes used in the mRNA analysis was set as the background; inferred electronic annotations were excluded; significance threshold was set to gSCS (>0.01); pathways that have a term size more than 350 were excluded; and Gene Ontology (BP) and REACTOME databases were used as the source, which was subsequently visualized in Cytoscape (v3.8.0; ref. 133) using the EnrichmentMap App (v3.3; ref. 134).

### Association of ETS and NKX3-1 Status with Mutation Densities

Patients were divided into four groups based on ETS mutation (positive or negative) and *NKX3-1* (neutral or loss) status. Then, the two-way ANOVA was used to investigate the main effects and interaction between each mutation density (log_10_-transformed) and the *ETS*/*NKX3-1* status. In addition, the Kruskal–Wallis tests were performed for each mutation density for the *ETS*/*NKX3-1* status. An FDR correction was used to adjust *P* values for multiple hypothesis testing. Drivers predicted by GISTIC and ActiveDriverWGS and also mutated in at least 40 patients (6% of this cohort) were tested using a generalized linear model to investigate the association between the drivers and the *ETS*/*NKX3-1* status. Drivers that showed significant main effects after the FDR correction (*Q <* 0.1) were further analyzed using the proportion test to investigate whether there was a difference in the proportion of patients with a driver relative to the *ETS*/*NKX3-1* status. FDR correction was used to adjust *P* values for multiple-testing.

### Analysis of Driver Co-occurrence and Mutual Exclusivity

Hypergeometric tests were used to assess whether driver mutations were statistically significantly co-occurring or mutually exclusive across the patient cohort. Only driver mutations present in at least 15 patients were tested. Two driver mutations were classified as co-occurring if there were more samples observed having both than expected by chance *Q* < 0.05. Pairs of somatic driver mutations were deemed mutually exclusive if there were fewer samples observed than expected by chance and *Q* < 0.05. Power was estimated by calculating the *P* value of the most extreme case to assess what the minimum possible *P* value would be given the observed driver counts. For mutually exclusive associations, the most extreme case was zero patients with both drivers. For co-occurring driver mutations, the most extreme case is all patients with the less frequent event having also having the more frequent one.

### Integrated Molecular Patient Subtypes

Patient subtypes were created by clustering all 243 driver regions using ConsensusClusterPlus (v1.8.1) after merging drivers of the same mutation type with Spearman’s ρ > 0.8. Drivers were merged by taking the union of their mutation sets. Clustering was performed with 2,000 iterations of hierarchical clustering and 80% subsampling of drivers and patients. Euclidean distance and a seed of 17 were used with Ward’s method for hierarchical clustering. Patients were further ordered within each subtype by clustering them based on genes that exhibit the highest proportion of driver events in each subtype using the Diana method.

### Correlates of Mutational Density

Correlations between measures of mutational density were calculated using Spearman’s ρ. *P* values were adjusted for multiple testing using the FDR method. Correlations between mutational density measures and clinical covariates were calculated using Spearman’s ρ. *P* values for associations between clinical variables with discrete values and mutational density measures were from a Kruskal–Wallis test. To account for differences in tumor and normal depth of sequencing coverage, a linear model was fit for each mutational density measure, and adjusted values were used in further analysis. A Kruskal–Wallis test was used to measure the association between each density measure and clinical variables, including age at diagnosis, pretreatment PSA, and T category. *P* values were adjusted for multiple testing. To determine clusters of driver genes, the percentage of tumors containing each mutation was calculated across different clinical variables, including age at diagnosis, pretreatment PSA, and T category. Continuous clinical variables age and PSA were discretized. Consensus clustering (ConsensusClusterPlus v1.38.0) was performed with a maximum of 20 clusters. Clustering was performed with 100,000 iterations of hierarchical clustering and 80% subsampling of drivers. Euclidean distance was used with Ward’s method for hierarchical clustering. Seven clusters were chosen based on the relative change in area under the cumulative distribution function (Supplementary Fig. S5E).

### Analysis of Progression across the ISUP GGs

A Kruskal–Wallis test was used to test the association between each mutation density and ISUP GG. *P* values were adjusted with the Bonferroni method. Pearson’s *χ*^2^ test was used to test for univariate associations with ISUP GG and clinical variables, including age at diagnosis, pretreatment PSA, and T category. To determine clusters of driver genes, the percentage of tumors containing each mutation was calculated across GGs. The two-way ANOVA was used to test for associations between the mutational frequency of genes across the ISUP GG. Consensus clustering (ConsensusClusterPlus v1.38.0) was performed, evaluating a maximum of 20 clusters. Clustering used 100,000 iterations of hierarchical clustering and 80% subsampling of drivers. Euclidean distance was used with Ward’s method of hierarchical clustering. The statistical power of each mutational density measure was calculated using the k-sample rank test under the Lehmann alternative hypothesis, in which the relative average of each ISUP GG was relative to GG 1 (clinfun v1.0.15). Associations between the ISUP GGs and the IMSs were tested using the Fisher exact test.

### Survival Analysis in Driver Regions Associated with Clinical Features

Cox proportional hazard models were fitted with the R package survival (v3.2–10) comparing the rate of biochemical relapse in patient samples with a mutation with those without. Only driver regions that were associated with clinical features were tested, and only when the driver region was observed in at least three samples. Adjusted models were also fit and adjusted for the clinical features (i.e., age, pretreatment PSA, ISUP GG, and T category) that driver region was associated with. *P* values were Benjamini–Hochberg false discovery–corrected.

### Discovery Patient Cohort

The germline-somatic discovery patient cohort included 427 patients of European descent, including 276 previously published samples: 83 were previously published in Wedge and colleagues (51), 50 in Baca and colleagues (25), 7 in Berger and colleagues (27), and 11 in Weischenfeldt and colleagues (26). Genetic ancestry was determined by calculating genetic distance to well-defined populations from the 1000 Genomes Project according to Heinrich and colleagues (106).

### Germline SNP Detection in the Discovery Cohort

We extended the “germline variant detection” pipeline from above with the following steps. Individual VCFs were merged using BCFtools (v.1.8), assuming SNPs not present in an individual VCF were homozygous reference. The MAF in the discovery cohort of all SNPs within the merged VCF was calculated and filtered to retain SNPs with MAF >0.01 based on the discovery cohort (nSNPs = 10,058,344). Next, all patients were re-genotyped using the GATK (v.4.0.2.1) at these sites to produce gVCFs (i.e., with option -ERC GVCF). Individual gVCFs were merged using GenomicsDBImport and joint genotyping was run using GenotypeGVCFs. Finally, SNPs were recalibrated using VariantRecalibrator and ApplyVQSR. We determined pathogenic variants within NCCN prostate cancer predisposition genes based on “pathogenic” or “likely pathogenic” annotations in ClinVar and ensuring more than one submitter (i.e., review status ≥2/4 stars).

### Recurrent Somatic Drivers for dQTL Analysis

We considered a set of 17 somatic drivers with a frequency of ≥5% in the discovery cohort that has been previously reported in localized prostate cancer: 11 CNA losses (seven trunk and four branch), 3 CNA gains (two trunk and one branch), 1 fusion (the recurrent T2E fusion between *TMPRSS2* and *ERG*), and 2 SNVs (26, 27, 29, 51, 85–89). CNAs represented by genes may be arm-level chromosome aberrations, such as loss of *NKX3-1* which often represents loss of the p-arm of chromosome 8 (29, 36). For a full definition of each somatic driver, refer to Supplementary Tables S1–S3.

### dQTL Discovery: Risk SNP dQTLs

The 147 SNP PRSs generated by Schumacher and colleagues (16) were first considered for dQTL discovery. Of the 147 SNPs, 135 had a MAF >0.05 in the discovery cohort. All 135 SNPs were tested for association with all 17 somatic drivers using a logistic regression model correcting for the first five genetic principal components, age, and mutation burden. *P* values were adjusted for multiple-hypothesis testing using Benjamini–Hochberg false discovery correction. Significance was defined as *Q* < 0.1.

### dQTL Discovery: Linear Local dQTLs

Local dQTLs were first defined based on the linear orientation of the genome. Considering each somatic event could be defined by a single gene, germline SNPs within ±500 kbp of the affected gene were interrogated for their association with the somatic event. Associations were quantified using a logistic regression model correcting for the first five genetic principal components, age, and the somatic mutation burden (i.e., PGA when testing CNAs and SNV mutation density when testing SNVs). Haplotype blocks within the defined linear local region were calculated considering the definition by Gabriel and colleagues (135), and a Bonferroni threshold considering α = 0.1 was used to determine significance. We selected a significance threshold of α = 0.1 to reduce false negatives in our discovery given its relatively small size. All discovered dQTLs were subsequently tested in an independent replication cohort to identify false positives.

### dQTL Discovery: Spatial Local dQTLs

Local dQTLs were defined taking into consideration the three-dimensional structure of DNA. The term spatial local was defined as regions of the DNA, outside ±500k bp around the affected gene, that loop to interact with the driver gene. First, these regions were defined by RAD21 ChIA-PET profiling in LNCaP and DU145 cell lines (61) and RNAPII ChIA-PET profiling in LNCaP, DU145, VCaP, and RWPE-1 cell lines (60). Coordinates of driver genes were overlapped with peak anchor regions using BEDtools. Based on an interaction map, peak anchors paired with driver gene–overlapped peaks were defined as interacting regions. Similar to linear local dQTLs, associations were quantified using a logistic regression model correcting for the first five genetic principal components, age, and the somatic mutation burden. Again, haplotype blocks within the defined spatial local region were calculated considering the definition by Gabriel and colleagues (135), and a Bonferroni-adjusted threshold at α = 0.1 was used.

### dQTL Discovery: Enhancer Local dQTLs

Spatial local regions were defined based on HiChIP H3K27ac profiling in LNCaP cell lines. HiChIP was conducted as reported previously. Again, associations were quantified using a logistic regression model correcting for the first five genetic principal components, age, and the somatic mutation burden, and haplotype blocks within the defined enhancer local region were calculated considering the definition by Gabriel and colleagues (135), and a Bonferroni threshold considering α = 0.1 was used to determine significance.

### Prostate Cancer dQTL Replication

Individuals of European descent, as determined by Yuan and colleagues (136), from The Cancer Genome Atlas (TCGA) PRAD were used as a replication cohort (13). As described previously (45), concordance between SNP6 microarray (SNP6) genotypes and whole-exome sequencing of blood sample genotypes was evaluated. Only samples with >80% concordance were retained (*n* = 412 samples). Genotypes were imputed using the Michigan Imputation Server pre-phasing using Eagle (v2.4; ref. 137), imputation using Minimac4 (138), and the Haplotype Reference Consortium (release 1.1) panel (139). A final list of 40,401,582 SNPs was then available for validation studies. SNV and CNA calls based on the hg19 reference genome were downloaded from GDC Legacy Archive (https://portal.gdc.cancer.gov/legacy-archive/search/). T2E fusions for TCGA samples were identified using FusionCatcher (v.0.99.7c; bioRxiv 2014.11.19.011650). A second cohort of 140 Australian men with localized prostate cancer was used to supplement the replication cohort. All men were of European descent, as determined according to Heinrich and colleagues (106). All patients had blood and tumor WGS processed with the same pipelines as the discovery cohort, including CNA timing. Similar to the discovery cohort, germline SNPs were identified using the GATK (v3.4.0–3.7.0; ref. 140). First, HaplotypeCaller was run on the normal and tumor BAMs together, followed by Variant Recalibration and ApplyRecalibration, following GATK best practices. Germline SNPs were filtered for somatic and ambiguous variants that had more than one alternate base.

### Pan-Cancer dQTL Replication

We leveraged the PCAWG (ref. 33) to test the replication of dQTLs in other cancer types, using germline VCFs and somatic CNA calls from PCAWG from the data coordination center (DCC) (https://dcc.icgc.org/releases/PCAWG/). We considered only adult cancers with >100 samples: breast, ovarian, pancreatic, and liver cancers. Next, we only considered patients of European ancestry which resulted in 134 breast, 91 ovarian, 116 pancreatic, and 0 liver patients with cancer. Thus, we did not consider liver cancer in replication analysis. We tested somatic events with a recurrence rate ≥5% in each cancer type.

### dQTL Replication Statistical Analysis

#### Overview

dQTLs with available somatic profiling and germline genotyping were tested in the replication cohort. TCGA does not have WGS, so the evolutionary timing of CNAs could not be determined in these patients. Thus, dQTLs involving CNAs were tested in TCGA without considering trunk versus branch classifications. As a result, there were significant differences in the proportion of cases and controls between the discovery and replication cohorts (Supplementary Table S1). T2E calls for TCGA samples in the replication cohort were based on RNA-seq alone compared with the rest of the samples which considered DNA sequencing or the union of DNA sdquencing and RNA-seq when available. dQTLs in all replication cohorts were tested using the same logistic regression model as used in discovery, correcting for the first five genetic principal components, age, and the total burden of somatic mutation type being tested (i.e., PGA or SNV mutation density). dQTLs were considered to have replicated if FDR <0.1 and sign[log(OR_discovery_)] = sign[log(OR_replication_)].

#### Replication of dQTLs in the ICGC EOPC

We identified nine dQTLs that were associated with somatic events with a recurrence rate ≥5% in the early onset prostate cancer (EOPC-DE) cohort and had concordant ORs in the discovery and replication cohorts. The candidate SNPs were studied across 238 patients with prostate cancer with European ancestry from the ICGC EOPC-DE cohort (63). Germline SNP genotyping and quality control was performed as previously described (141). Association between germline SNP genotypes and the presence of somatic mutation was performed using logistic regression models in Python (stats package v0.11.1) correcting for the first five principal components, age, and mutational burden.

#### Replication of dQTLs in Hartwig Medical Foundation Metastatic Prostate Cancer

We replicated dQTLs on the external Center of Personalized Cancer Treatment/Hartwig Medical Foundation (CPCT-02/HMF) dataset under data requests DR-071 and DR-208 (64). This is an extension of the metastatic prostate cancer cohort (*n* = 394 distinct patients) as previously described by van Dessel and colleagues (142). To select patients of (predominantly) European descent, we utilized the established set of ancestry markers from the EUROFORGEN Global AIM-SNP set (143) which consisted of 128 biallelic and triallelic germline markers and 934 respective reference samples of African, East Asian, European, Native American, and Oceanian ancestry. For these ancestry markers, we determined the respective germline genotype (0/0, 0/1, 1/1, 0/2, 1/2, or 2/2) within all distinct patients within the CPCT-02/HMF dataset. Subsequently, we performed a principal component analysis (PCA) on the combined dataset of genotypes from the CPCT-02/HMF dataset and reference samples. As input for the PCA, genotypes were converted into six numerical categories (0–5) and zero centered and scaled during the PCA. To determine the putative ethnicity of the CPCT-02/HMF patients, we performed a K-Means clustering (*k* = 5, Hartigan and Wong algorithm on 50 random sets and 10,000 iterations) on all principal components (i.e., ancestry markers) as derived on the combined genotype matrix of the reference samples and the CPCT-02/HMF dataset. From this analysis, we selected the distinct CPCT-02/HMF patients clustering within the European descent reference cluster (*n* = 384). For these 384 European CPCT-02/HMF metastatic patients, we determined the germline genotypes of the dQTLs (*n* = 19) and the presence of the respective somatic event within the tumor genome (somatic deletions of *CDKN1B*, *CHD1*, *RB1*, *TMPRSS2*, and/or *ZNF292*, amplifications of *NCOA2*, somatic mutations within the 3′ UTR of *FOXA1*, and genomic fusions of *TMPRSS2*-*ERG*). If multiple metastatic biopsies from the same patients were available (*n* = 43), the aggregation of respective somatic events within a patient was used to determine the presence of these somatic events. dQTLs were assessed within a logistic regression model correcting for the first five genetic principal components (based on the ancestry markers), age, and mutational burden.

#### Replication of dQTLs in PROFILE

The Dana-Farber Cancer Institute prospective cohort (PROFILE) was collected with informed consent: 490 unrelated men of European descent with prostate cancer (91 with metastatic disease and 399 primary or local tumors). All samples underwent targeted sequencing on the OncoPanel platform with three panel versions that targeted the exons of 275, 300, and 447 genes, respectively. Genotypes were imputed from off-target reads using STITCH (v.1.5.3; ref. 144). To determine genetic ancestry, reference principal components were computed by SNPweight tools in HapMap populations of European, West African (Yoruban), and East Asian (Chinese) ancestry (145). In the PROFILE cohort, imputed dosages for variants with INFO >0.4 and MAF >0.01 were projected in the same PCA space using the PLINK2 “–score” function. The mean principal component along both the West African–European cline and the East-Asian–European cline was computed for all individuals who self-reported as White. Individuals within ± two SDs were retained. Samples were filtered for relatedness using a GRM matrix with a 0.05 cutoff. SNPs were filtered to ensure Hardy–Weinberg equilibrium *P* value >0.001, MAF >0.05, and INFO >0.4. If the tag dQTL was not genotyped in the PROFILE cohort, a proxy SNP was selected by maximizing the product of the INFO, R^2^, and 1000 Genomes European MAF using LDlinkR (146). Finally, associations were tested using a logistic regression in PLINK2 with the first five genetic principal components, tumor purity, panel version, age, and PGA as covariates.

### dQTL Meta-analysis

Effect sizes and SEs of dQTL associations in the discovery, replication, HMF, EOPC, and PROFILE cohorts were combined using a restricted maximum likelihood model as implemented in the metafor R package (v3.0.2).

### Germline Methylation (meQTL) Associations

To assess the effect of dQTLs on the tumor methylome, the 16 concordant tag dQTLs were evaluated for local meQTLs, defined as probes ±500 kbp around the SNP, using a linear regression correcting for the first five genetic principal components and age. *P* values were adjusted for multiple-hypothesis testing using Benjamini–Hochberg false discovery correction. Significance was defined as Q < 0.10. Significant meQTLs were next replicated in the TCGA cohort using the same linear regression modeling, in which replication was defined as Q_replication_ < 0.10 and sign(β_replication_) = sign(β_discovery_). Replicated meQTLs were tested for tumor specificity considering patients that had matched tumor/reference methylation profiling (*n* = 50). Tumor specificity was defined as Q_tumor_ < 0.10 and Q_reference_ > 0.10 or sign(β_tumor_) ≠ sign(β_reference_) using the same linear regression model. To assess enrichment of meQTLs, we generated a null distribution of the number of SNPs involved in a replicated meQTL and the number of replicated meQTLs. We randomly sampled 16 SNPs from the total list of SNPs evaluated as a local dQTL against any driver. We identified and replicated local meQTL ±500 kbp around each of the 16 random SNPs using the same methods as the dQTL–meQTL analysis. We calculated the number of SNPs involved in a replicated meQTL as well as the total number of replicated meQTLs. We repeated this 1,000 times. *P* values were calculated as 1 – the fraction of iterations more dQTLs were involved in a replicated meQTL than random SNPs or 1 – the fraction of iterations dQTLs were involved in more replicated meQTLs than random SNPs.

### Germline–Chromatin Associations

Peak BED files for H3K27ac (*n* = 92), H3K27me3 (*n* = 76), AR (*n* = 88), and H3K4me3 (*n* = 56) were used from an independent cohort of 94 patients with localized prostate cancer (GSE120738; ref. 67). dQTLs overlapping each target were identified using the downloaded BED files. We considered a dQTL overlapping if any of the SNPs in its haplotype block overlapped the target. A second cohort of 48 patients with localized prostate cancer was additionally profiled, as described previously (45). Briefly, both adenocarcinoma and nonmalignant prostate tissue from each patient was subjected to ChIP-seq for H3k27ac (*n* = 48), H3k4me2 (*n* = 6), H3k4me3 (*n* = 4), FOXA1 (*n* = 10), and HOXB13 (*n* = 9) and blood samples were genotyped for germline SNPs followed by imputation using the HRC panel (139). Sites of allelic imbalance in the ChIP-seq peaks were identified by first correcting for mapping bias using the WASP pipeline (147), peak calling using MACS2, and finally testing for allele-specific signal using GATK ASEReadCounter (140) and a beta-binomial test. Each test was performed once for samples from normal, tumor, or both, as well as a test for difference in imbalance between tumor and normal. Peaks were considered “imbalanced” in each of these four test categories if any of the SNPs tested for that peak exhibited allele-specific signal at a 5% FDR. Finally, we tested the overlap of dQTLs with published ChIP-seq data from LNCaP, PC3, 22Rv1, VCaP, and RWPE-1 cell lines (68–81). If multiple target:treatment pairs existed, the median number of overlapping SNPs was used.

### Germline–RNA (eQTL) and Germline–Protein (pQTL) Associations

Next, the 16 SNPs involved in the 23 concordant dQTLs were tested for their effect on the transcriptome (128). We evaluated local eQTLs, defined as genes ±500 kbp around the SNP. mRNA abundance TPM values for each gene were rank inverse normalized. eQTLs were tested using a linear regression model correcting for the first five genetic principal components, age, and 10 PEER (148) factors to adjust for noise in the RNA-seq data. *P* values were adjusted for multiple-hypothesis testing using the Benjamini–Hochberg false discovery correction. Nominally significant eQTLs were considered for pQTL discovery using protein abundances from mass spectrometry as described previously (127). pQTLs were tested using a linear regression model correcting for the first five genetic principal components, age, and 10 PEER factors to adjust for noise in the mass spectrometry data.

### dQTL Clinical Associations

Germline SNPs in dQTLs were associated with clinical characteristics, including PSA, ISUP GG, T category, age at diagnosis, and biochemical recurrence. PSA and age were tested using a linear regression model, correcting for the first five genetic principal components. The PSA model was also corrected for age. T category was tested using a logistic regression model comparing T2 to ≥ T3, correcting for the first five genetic principal components and age. ISUP GG was tested by using an ordinal linear regression model, correcting for the first five genetic principal components and age. Each clinical outcome was independently corrected for multiple hypothesis testing using the Benjamini–Hochberg false discovery correction. Survival analysis with biochemical recurrence was tested using a Cox proportional hazards model. Three genetic models (dominant, recessive, and codominant) were tested, and the model with the lowest AIC was reported. Kaplan–Meier curves were plotted. HR was adjusted for primary treatment.

### dQTL Somatic SNV Enrichment

For each of the 16 SNPs involved in the high-confidence dQTLs, we assessed whether the somatic SNV mutation burden ±10 Mbp of the dQTL was higher than expected using ActiveDriverWGS (38) *P* values were adjusted for multiple hypothesis testing using Benjamini–Hochberg false discovery correction.

### Ancestral VAF Bias

VAFs in European (*n* = 7,718), African (*n* = 4,359) and East Asian (*n* = 780) populations for the 16 dQTL SNPs were extracted from gnomAD (v2.1.1; ref. 149). Allele frequencies in African and East Asian populations were compared with the European population using the Fisher exact test, and the FDR was applied across all 16 SNPs in each comparison separately. As a control, North-West European VAFs were compared again other non-Finnish European VAFs using the Fisher exact test. These two European populations were chosen because they had the largest sample number in gnomAD. To estimate the proportion of ancestral differences in T2E and *FOXA1* mutation frequency explained by dQTLs, we compared the ORs of ancestry–somatic associations and dQTL ORs multiplied by normalized VAF differences between the two ancestry groups. For example, we estimated OR_European versus African_ (T2E) = 5.00 and OR_European versus African_ (*FOXA1* SNVs) = 0.50 based on Huang and colleagues (86) and Lindquist and colleagues (88) compared with the somatic driver frequency in the discovery cohort. We estimated OR_European versus East Asian_ (T2E) = 7.47 and OR_European versus East Asian_ (*FOXA1* SNVs) = 0.07 based on Li and colleagues (87) compared with the somatic driver frequency in the discovery cohort.

### dQTL Power Analysis

Power was estimated based on the non-centrality parameter of the *χ*^2^ statistic under the alternative hypothesis using the R package gwas-power (https://github.com/kaustubhad/gwas-power). Power was calculated for varying MAF and effect size values considering sample sizes reflective of somatic driver frequencies 0.05, 0.20, and 0.50 in the discovery cohort. To estimate the number of non-detected dQTLs, discovered dQTLs were binned based on their MAF, effect size, and somatic driver frequency. The number of detected dQTLs in each bin was divided by the corresponding power to estimate the total number of expected dQTLs. We subtracted the discovered dQTLs from expected dQTLs to estimate the number of nondetected dQTLs.

### Assessment of Skew of dQTL *P* Value Distributions

To determine whether dQTL *P* value distributions were significantly skewed to small *P* values more than expected by chance alone, a null distribution for each analysis (i.e., linear local and spatial local) and each somatic driver was generated by permuting the somatic driver labels. That is, for a single somatic event, patients were randomly assigned whether or not they had the somatic event while maintaining the true frequency of the event in the cohort. Next, both linear and spatial local dQTL discovery analyses were conducted as described above with the permuted somatic driver labels. The skew of the −log_10_*P* value distribution was calculated and compared with the true distribution. *P* values were calculated by considering the number of permutation iterations that had skew > real skew divided by the number of iterations performed. One thousand iterations were performed for each somatic driver. To supplement these analyses, we also estimated the proportion of null *P* values in the *P* value distributions for linear, spatial, and enhancer dQTLs for the top five most recurrent somatic mutations using the pi0est() function in the qvalue R package (v2.18.0).

### Data Visualization

Visualizations were generated in the R statistical environment (v3.3.1–4.1.2) using the lattice (v0.24–30), latticeExtra (v0.6–28), and BPG package (v5.6.23–7.0.3; ref. 150) along with pdfTeX (3.14159265-2.6-1.40.16), Gliffy Diagram for Jira, Inkscape (v0.91), and GIMP (v2.8). mtSNV visualization was performed using Circos (v0.69–6; ref. 151). Figures 1A, 3A, and 7G were created using BioRender.com under a Creative Commons (CC-BY) license.

### Data Availability

All Canadian raw sequence data and variant calls are available on the European Genome-Phenome Archive (EGA) under accession EGAS00001000900 (https://www.ebi.ac.uk/ega/studies/EGAS00001000900) and Australian raw sequence data and variant calls are available on the EGA under accession EGAS00001003088 (https://www.ebi.ac.uk/ega/studies/EGAS00001003088). Canadian mRNA data are available on Gene Expression Omnibus (GEO) under accession GSE84043 (https://www.ncbi.nlm.nih.gov/geo/query/acc.cgi?acc=GSE84043). Baca WGS data are available on dbGaP under accession phs000447.v1.p1 (https://www.ncbi.nlm.nih.gov/gap/?term=phs000447.v1.p1). Berger WGS data are available on dbGaP under accession phs000330.v1.p1 (https://www.ncbi.nlm.nih.gov/gap/?term=phs000330.v1.p1). Weischenfeldt WGS data are available on the EGA under accession EGAS00001000400 (https://www.ebi.ac.uk/ega/studies/EGAS00001000400). TCGA WGS data are available at Genomic Data Commons Data Portal (https://portal.gdc.cancer.gov/projects/TCGA-PRAD). French ICGC WGS data are available on the EGA under accession EGAD00001003115 (https://www.ebi.ac.uk/ega/datasets/EGAD00001003115). UK ICGC WGS data are available on the EGA under accession (https://www.ebi.ac.uk/ega/datasets/EGAD00001001116). Processed germline variant calls are available through the ICGC Legacy SFTP server (Host: icgc-legacy-1417 sftp.platform.icgc-argo.org, Port: 2222) with approved DACO access (https://docs.icgc-1418argo.org/docs/data-access/icgc-25k-data). Detailed information on access to these data is available at: https://docs.icgc-argo.org/docs/data-access/icgc-25k-data. Methylation data are available in GEO under accession GSE84043. Primary samples’ ChIP-seq data were retrieved from GEO under accession GSE120738.

## Supplementary Material

Supplementary Table 1 Supplementary Table 1 shows a feature-by-patient summary matrix. For each patient, this table provides version information of bioinformatics tools, summary sequencing statistics, mutational density metrics, clinical information and driver mutation status for all drivers detected using GISTIC and ActiveDriverWGS as or identified in Armenia and colleagues 2018. Two additional tables are provided for the clinical and mutation data for the discovery and replication cohorts of dQTL discovery.

Supplementary Table 2 Supplementary Table 2 displays results from driver selection, driver groupings and driver associations.

Supplementary Table 3 Supplementary Table 3 illustrates driver co-occurrence analysis, driver clusters, and associations of drivers with clinical features

Supplementary Table 4 Supplementary Table 4 shows driver selection for dQTL nomination and prevalence of drivers in cohorts

Supplementary Table 5 Supplementary Table 5 displays summary statistics from PRS and HOXB13 associated with somatic drivers. β and P-value from logistic regression correcting for five genetic principal components, age and somatic mutation burden. FDR = false discovery rate.

Supplementary Table 6 Supplementary Table 6 displays the number of dQTLs identified for each somatic driver in each analysis strategy. Summary statistics from local dQTL associations. Statistics from logistic regression correcting for five genetic principal components, age and somatic mutation burden. OR = odds ratio; SE = standard error; L95 = lower 95% confidence interval; U95 = upper 95% confidence interval

Supplementary Table 7 Supplementary Table 7 provides summary statistics from distal dQTL associations

Supplementary Table 8 Supplementary Table 8 shows summary statistics of 16 SNPs associated with 23 concordant dQTLs across cohorts

Supplementary Table 9 Supplementary Table 9 summarizes the characterization of 16 SNPs associated with 23 concordant dQTLs.

Supplementary Table 10 Supplementary Table 10 lists dQTL SNPs identified as eQTLs in prostate tissue in GTEx.

Supplementary Table 11 Supplementary Table 11 reports percentages of cross-individual contamination for each sample and sequencing lane.

Supplementary Figures Supplementary Figures & Figure Legends. Supplementary Figure 1 | Cohort Structure and Analysis. Supplementary Figure 2 | CNA Evolution & Transcriptomic Effects. Supplementary Figure 3 | Properties of Driver Mutations. Supplementary Figure 4 | Pathway & Signature Analysis of Driver Genes. Supplementary Figure 5 | Patterns of Mutational Drivers. Supplementary Figure 6 | Molecular Correlates of Clinical Behavior. Supplementary Figure 7 | Heterogeneity of Driver-Clinical Associations. Supplementary Figure 8 | Cohort Characteristics and Risk dQTL Replication. Supplementary Figure 9 | Local dQTLs Discovery. Supplementary Figure 10 | Replication of dQTLs. Supplementary Figure 11 | Enrichment of Sub-threshold dQTLs. Supplementary Figure 12 | Molecular Characterization of dQTLs. Supplementary Figure 13 | Association of dQTL Risk SNPs with eQTL and IMS. Supplementary Figure 14 | Clinical Characterization of dQTLs.
