# Supplementary material for: The Germline and Somatic Origins of Prostate Cancer Heterogeneity
Source: Cancer Discov. 2025 Feb 13;15(5):988–1017. doi: 10.1158/2159-8290.CD-23-0882 (PMC12046336; doi:10.1158/2159-8290.CD-23-0882)
Supplement: Supplementary Figures — & Figure Legends. Supplementary Figure 1 | Cohort Structure and Analysis. Supplementary Figure 2 | CNA Evolution & Transcriptomic Effects. Supplementary Figure 3 | Properties of Driver Mutations. Supplementary Figure 4 | Pathway & Signature Analysis of Driver Genes. Supplementary Figure 5 | Patterns of Mutational Drivers. Supplementary Figure 6 | Molecular Correlates of Clinical Behavior. Supplementary Figure 7 | Heterogeneity of Driver-Clinical Associations. Supplementary Figure 8 | Cohort Characteristics and Risk dQTL Replication. Supplementary Figure 9 | Local dQTLs Discovery. Supplementary Figure 10 | Replication of dQTLs. Supplementary Figure 11 | Enrichment of Sub-threshold dQTLs. Supplementary Figure 12 | Molecular Characterization of dQTLs. Supplementary Figure 13 | Association of dQTL Risk SNPs with eQTL and IMS. Supplementary Figure 14 | Clinical Characterization of dQTLs. [file cd-23-0882_supplementary_figures_suppsf1.pdf]

Supplementary Figure 1

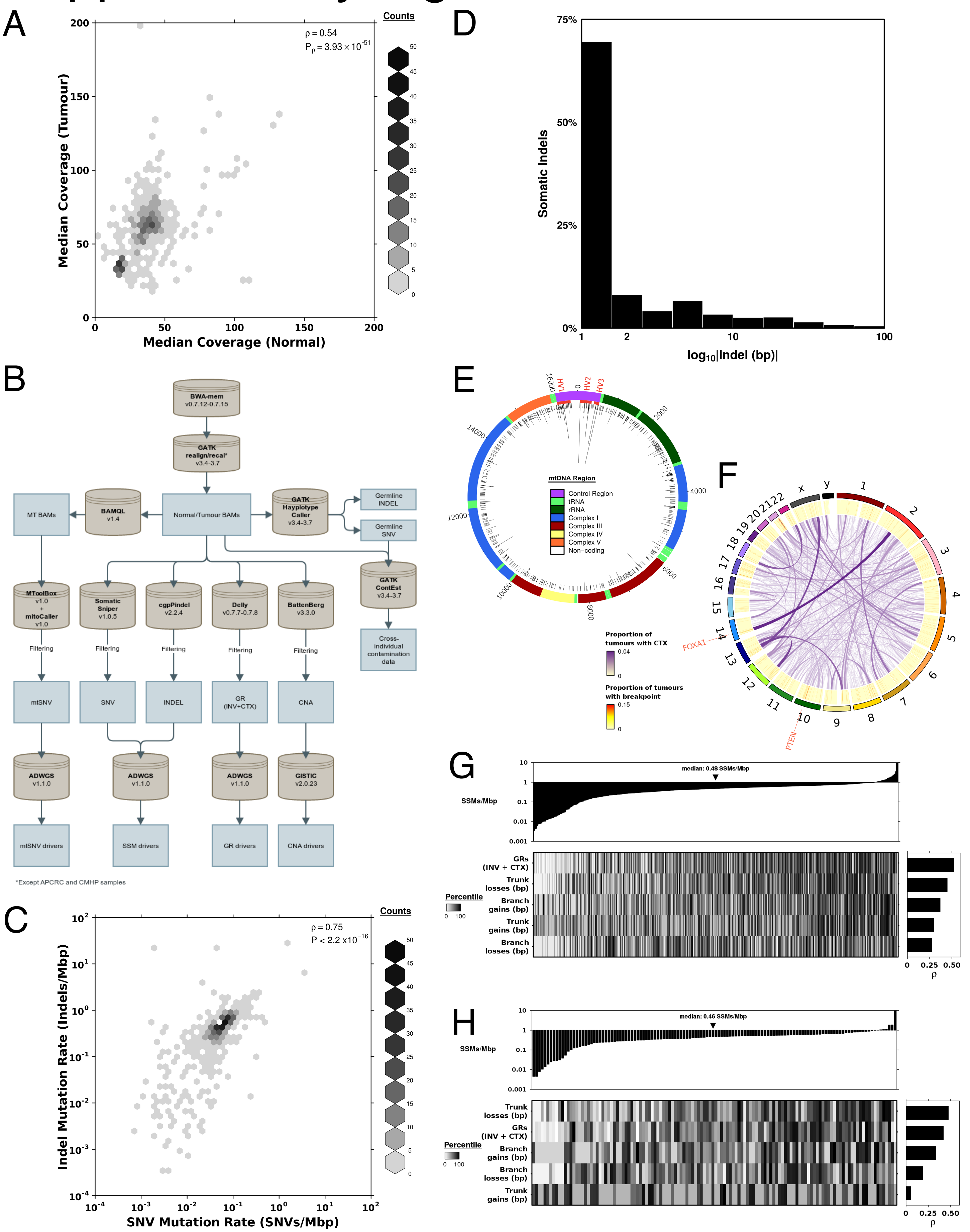

Supplementary Figure 1 | Cohort Structure and Analysis

A) The correlation of median normal and tumor median coverage using Spearman's ρ. **B)** Correlation between somatic SNV and Indel mutation rate using Spearman's ρ. **C)** Bioinformatics analysis pipeline, starting from WGS sequencing alignment to GRCh37, variant detection and mutational driver prediction. The details of each component are described in the Methods section. **D)** Distribution of somatic Indel lengths. **E)** The distribution of mtSNVs across the mitochondrial RSRS genome. The recurrence of mtSNVs is shown by the inner histogram. **F)** Circos plot of translocations across the entire cohort of 666 samples. **G)** For each type of mutational density, samples were percentile ranked, then ordered by the rate of somatic single mutations (SSMs; (SNVs + Indels)/Mbp). Mutational density measures were ordered by Spearman's ρ with SSM/Mbp, as shown in the barplot to the right. **H)** For ISUP Grade Group 2 patients with ≥60% tumor purity, samples were percentile ranked for each mutational density measure, then ordered by the number of SSMs/Mbp. Mutational density measures are ordered by Spearman's ρ with SSM (SNV + Indel) rate.

Supplementary Figure 2

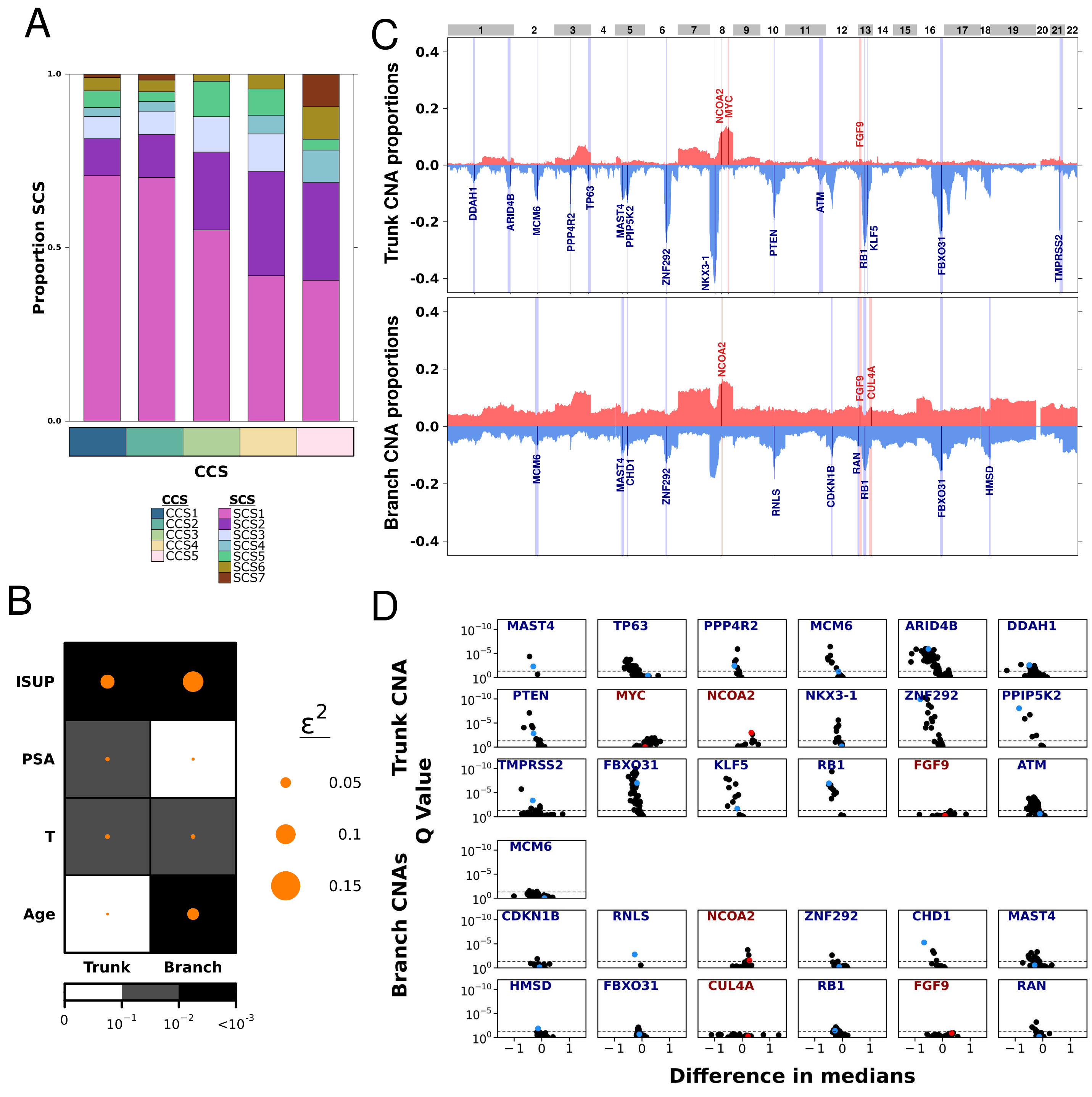

Supplementary Figure 2 | CNA Evolution & Transcriptomic Effects

A) Relative proportion of subclonal subtypes (SCS) for each clonal subtype (CCS). **B)** A summary of associations between CNA subtypes and clinical features. Dot size indicates effect size (ε^2) between each clinical feature and CNA subtype for clonal and subclonal CNA profiles. Background colors represent FDR adjusted Q-values from Kruskal-Wallis tests. **C)** The proportion of patients with gains and deletions for each gene. The positive direction shows the proportion of patients with a gain and the negative direction shows the proportion of patients with a deletion. The x-axis is genes ordered by genomic position. The boxes across the length of the plots are the significant regions from GISTIC analysis with gain regions in red and deletion regions in blue. The darker lines labeled with the gene are the genes selected from each GISTIC region. **D)** Each volcano plot shows CNA associated mRNA abundance differences for the genes in a GISTIC region. The selected gene for each region is the red/blue point. Blue gene labels indicate the comparison of mRNA abundance from patients with deletions to patients without. Red gene labels indicate the comparison of mRNA abundance from patients with gains to patients without. The top 3 rows are clonal (trunk) CNAs and the bottom 3 rows are subclonal (branch) CNAs. The Q-values are from Wilcoxon rank-sum tests. For each subfigure, CNAs in patients with subclonal PGA >80% were excluded.

Supplementary Figure 3

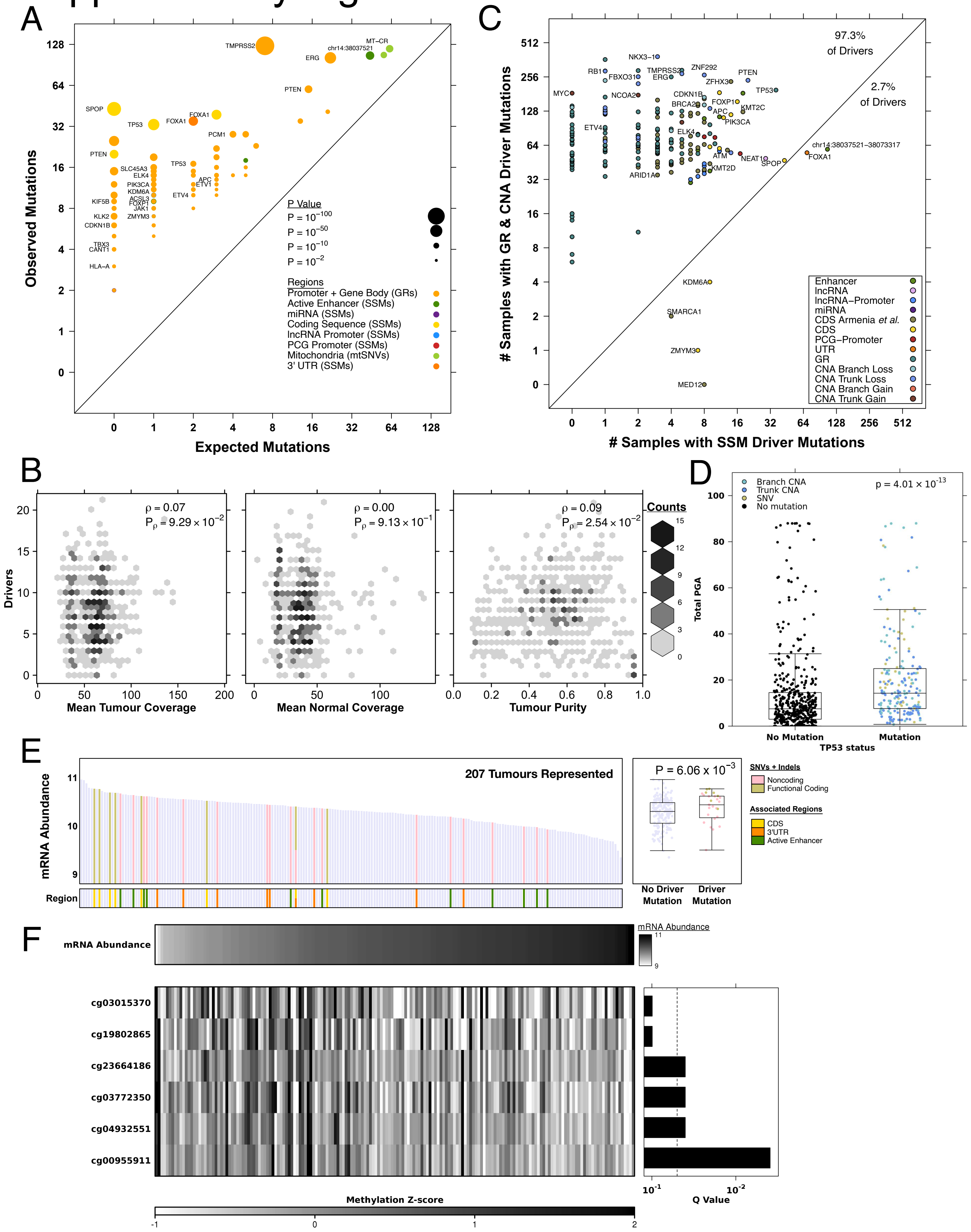

Supplementary Figure 3 | Properties of Driver Mutations

A) Observed *vs.* expected mutation counts for driver events discovered *via* ActiveDriverWGS (*i.e.* SNVs and Indels, GRs and mitochondrial SNVs). Observed is the actual number of patients (n = 666) with mutations; expected is the number of mutations expected by chance based on background mutation rates, region length and sequence composition. Dots are colored by genomic region and sized by P-value. **B)** The number of drivers per tumor is not correlated with sequencing coverage and only weakly correlated with tumor purity (based on estimation by Battenberg). Statistical significance was assessed using a Spearman's correlation. **C)** The number of samples harbouring a CNA or balanced GR in a driver region *versus* the number of samples harbouring a simple somatic mutation in 666 prostate cancer samples. Regions are colored by the primary type of somatic mutation in which driver discovery acquired statistical significance. **D)** There is a difference in total PGA (branch PGA + trunk PGA) between samples with a *TP53* mutation (either branch or trunk CNA or SNV) and those without. **E)** *FOXA1* mRNA abundances ($\log_2$) in 207 tumors. Barplot and boxplot are colored by mutation type, where non-coding mutations belong to the *FOXA1* 3' UTR or its associated enhancer. P-value is from a two-sided Wilcoxon rank-sum test **F)** *FOXA1* methylation probes associated with *FOXA1* mRNA abundance in samples without *FOXA1* mutations. *FOXA1* mRNA abundance is shown in the top bar, methylation levels are shown in the lower rows and the right bar shows the Q-value for association of methylation levels with median dichotomized mRNA abundance using two-sided Wilcoxon rank-sum tests.

Supplementary Figure 4

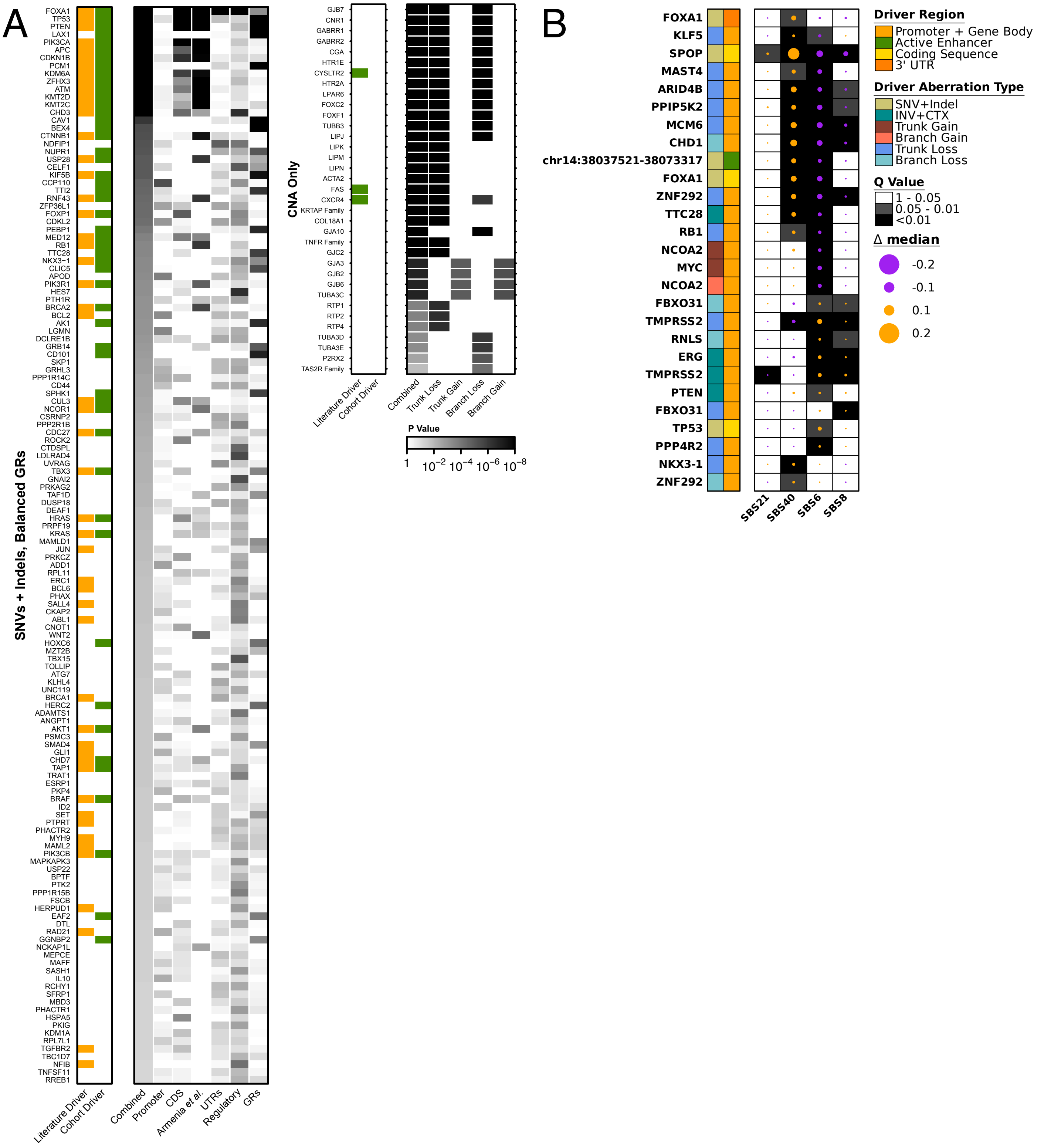

Supplementary Figure 4 | Pathway & Signature Analysis of Driver Genes

A) Heatmap of all genes contributing to pathway analysis (*i.e.* overlap of statistically significant genes and pathways). The combined contribution is given in the left column (Brown's method)(46). The covariate on the right indicates whether the driver gene is known from the literature or was univariately statistically significant in this study. **B)** A summary of associations between driver mutations and SBS signatures with mean signature activity > 5% including non-canonical SBSs (SBS6 and SBS21) which were not identified in the PCAWG study(48). Patients were dichotomized based on the presence of each driver mutation. Dot color and size show the median difference in signature activity. Background shading shows the Q-values from the Wilcoxon rank-sum test. Drivers and SBSs were ordered using hierarchical clustering constructed by Canberra distance with Single-link method.

Supplementary Figure 5

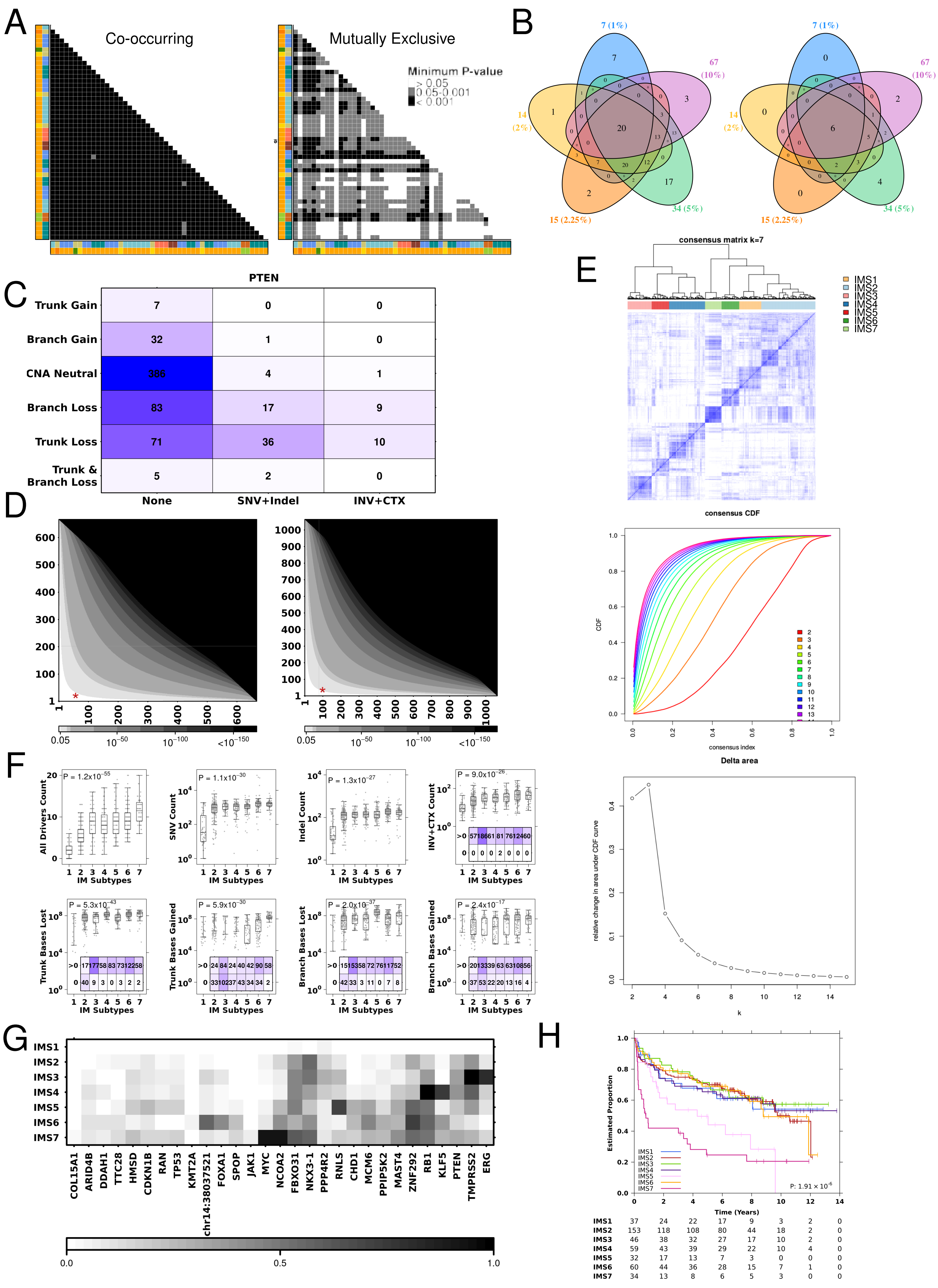

Supplementary Figure 5 | Patterns of Mutational Drivers

A) The minimum P-value (shading) achievable using a hypergeometric test for each possible pair of driver mutations, as in **Figure 4D**, for co-occurring *i.e*. maximum overlap (left) or mutually exclusive *i.e*. minimum overlap drivers (right). Note that $P < 0.001$ reflects $Q < 0.05$ in this cohort. **B)** The number of significant driver pairs co-occurring (left) and mutually exclusive (right) when different prevalence thresholds were used to filter the driver regions between analyses. Thresholds tested are greater than or equal to 1%, 2%, 5%, 10% of patients and 15 patients (~2.25%). **C)** Contingency table showing presence of CNAs, SNV+Indel and INV+CTX for *PTEN*. **D)** Minimum achievable P-value for mutual exclusivity comparisons given our cohort size of 666 patients (left) and 1,063 patients (right). Each axis is the count of one driver event. The red star is exactly at statistical significance for 1,063 patients, and reflects a comparison between *PTEN* SNV+Indel and *PTEN* CTX+INV at their frequency in this cohort. **E)** ConsensusCluster results. Top: consensus matrix. Middle: cumulative distribution functions for different cluster numbers. Bottom: change in area under cumulative distribution functions as a function of the number of clusters. **F)** Distribution of mutation densities within patient subtypes. Each plot shows mutational density on the y-axis and mutational subtypes (IMS1-IMS7) on the x-axis. P-values are from a Kruskal-Wallis test, and patients with no mutations of that type are not visualized in log-scale plots but are included in statistical analyses. Table inserts show the number of patients with no mutations *vs.* any mutations of that type; if no table insert is shown, all values were plotted. **G)** For each mutational subtype (row) and a subset of mutational driver events (columns), the shading shows the proportion of patients with that mutation present in that subtype. **H)** Kaplan-Meier plot showing the difference in biochemical relapse rate between the seven integrated molecular subtypes. P-value is from a log-rank test. For **A) B), F),** and **G)**, CNA drivers in patients with subclonal PGA >80% (reflecting likely subclonal whole-genome duplication) were excluded.

Supplementary Figure 6

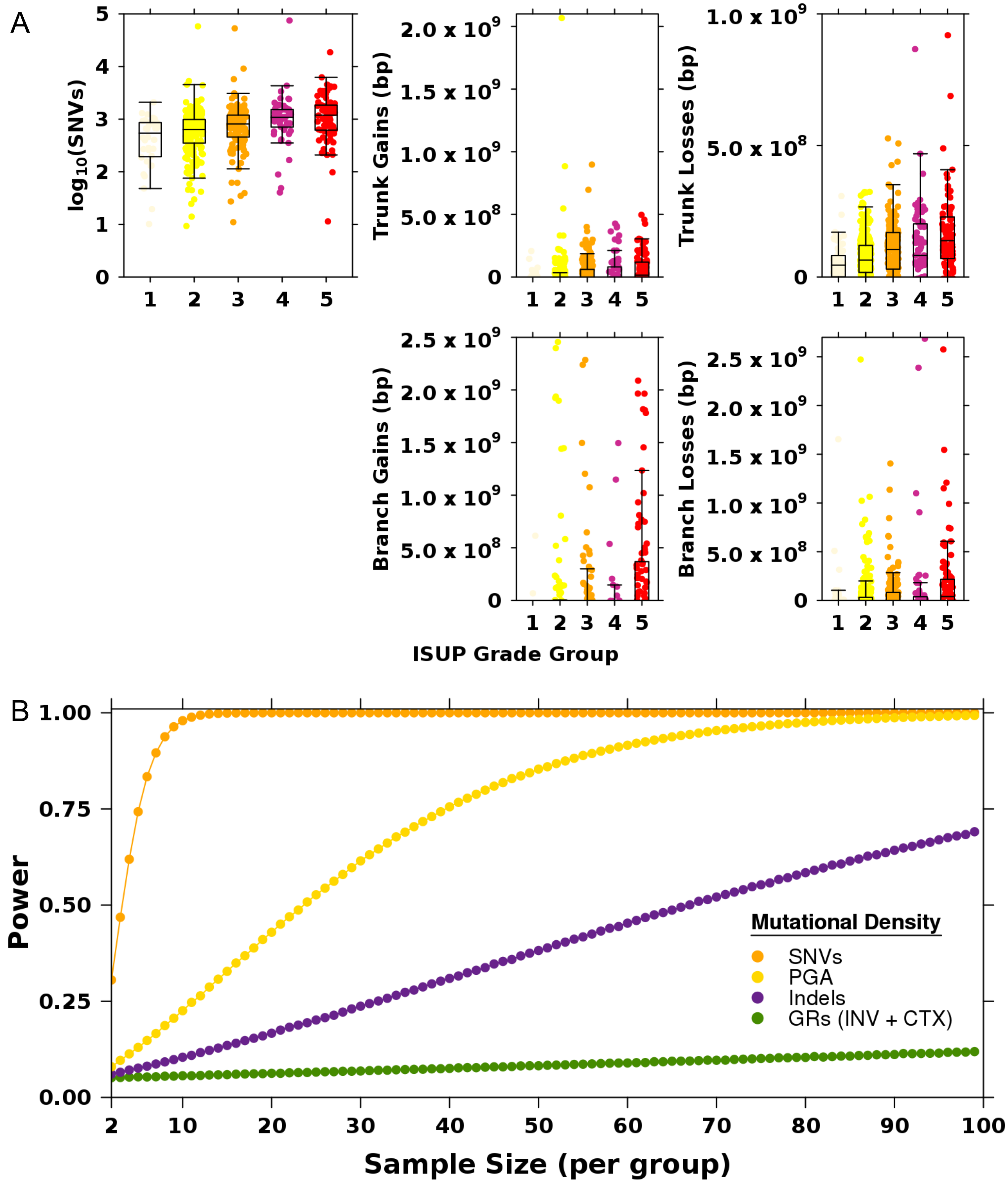

Supplementary Figure 6 | Molecular Correlates of Clinical Behavior

A) The number of SNVs, bases gained, and lost clonally, and bases gained and lost subclonally in each tumor, stratified by ISUP Grade Group. **B)** Statistical power for identifying associations of mutational density measure with ISUP Grade as a function of sample-size within each ISUP Grade Group, relative to GG 1.

Supplementary Figure 7

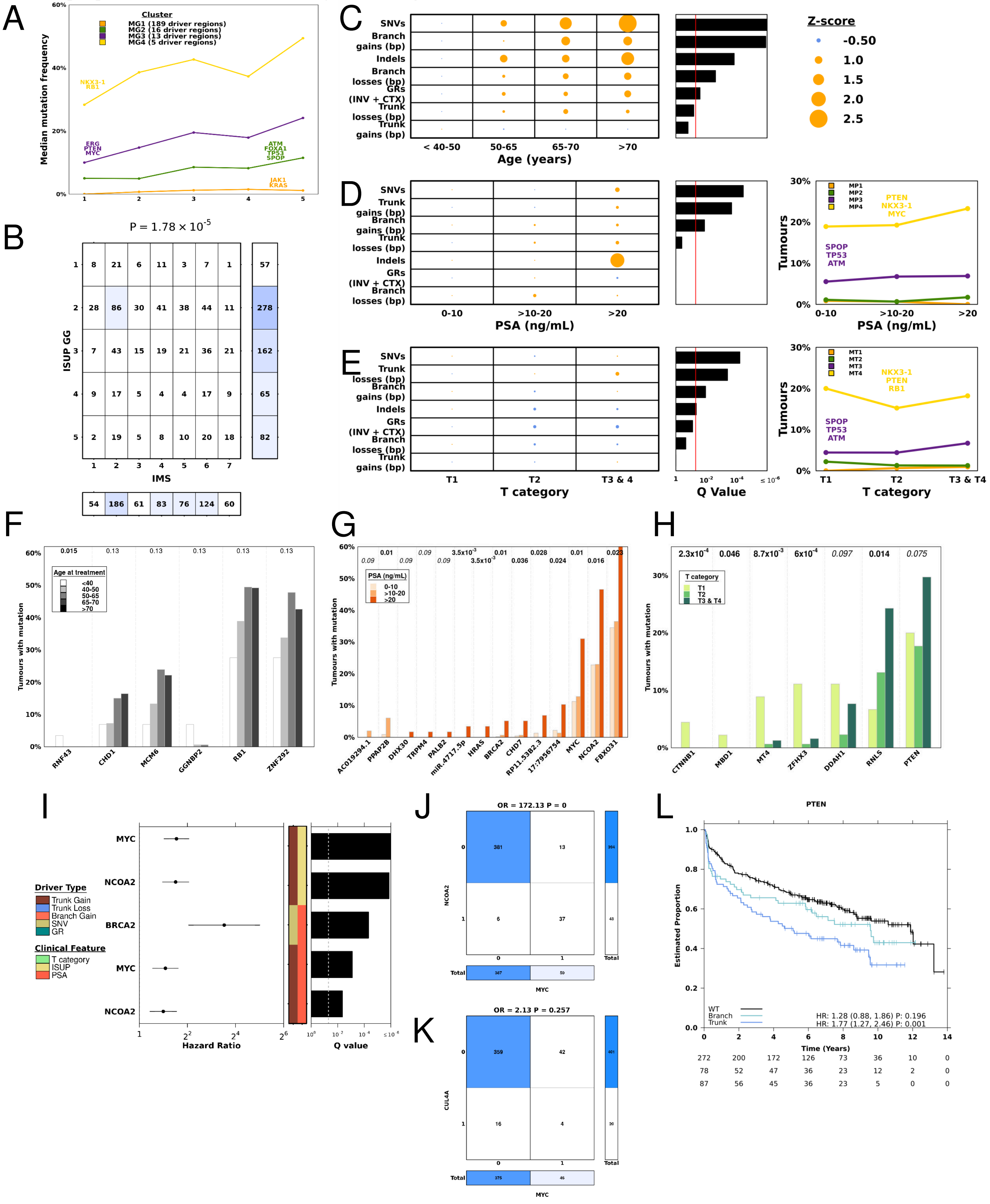

Supplementary Figure 7 | Heterogeneity of Driver-Clinical Associations

A) Consensus clustering identified four groups of genes with similar patterns of change across ISUP Grade Groups. For each gene cluster, the median mutation frequency for each ISUP Grade Group is shown, along with exemplar genes for each cluster. **B)** Contingency table of ISUP Grade Groups and the integrative molecular subtypes (IMS). **C)** A linear model was fit to relate each mutational density measure to age at diagnosis, using tumor and normal sequencing coverage as covariates. Dot size and color represents the effect-size for each group as a Z-score relative to patients under the age of 50. The barplot shows the FDR-adjusted P-value from a non-parametric Kruskal-Wallis test. **D)** As in **C)**, but for pre-treatment serum PSA concentration, relative to PSA ≤ 10 ng/mL. **E)** As in **C)**, but for tumor-extent at diagnosis (clinical T Category), relative to tumors categorized as T1. **F)** Genes whose median frequency is univariately associated with age at treatment, ordered by the percentage of samples with mutations in ISUP GG 5 tumors. FDR-adjusted P-values from Pearson's χ^2 test are shown. **G)** As in **F)**, but with genes associated with pre-treatment serum PSA concentration. **H)** As in **F)**, but for genes associated with T Category. **I)** Cox proportional hazard models were fit for driver regions and adjusted for the clinical feature they were associated with. Significant regions after FDR adjustment are shown, as well as the driver type and clinical feature the region was associated with. **J)** Contingency table of *MYC* and *NCOA2* gains. **K)** Contingency table of *MYC* and *CUL4A* gains. **L)** *PTEN* clonal but not subclonal losses were associated with biochemical relapse. For **F-L)**, CNA drivers in patients with subclonal PGA > 80% were excluded.

Supplementary Figure 8

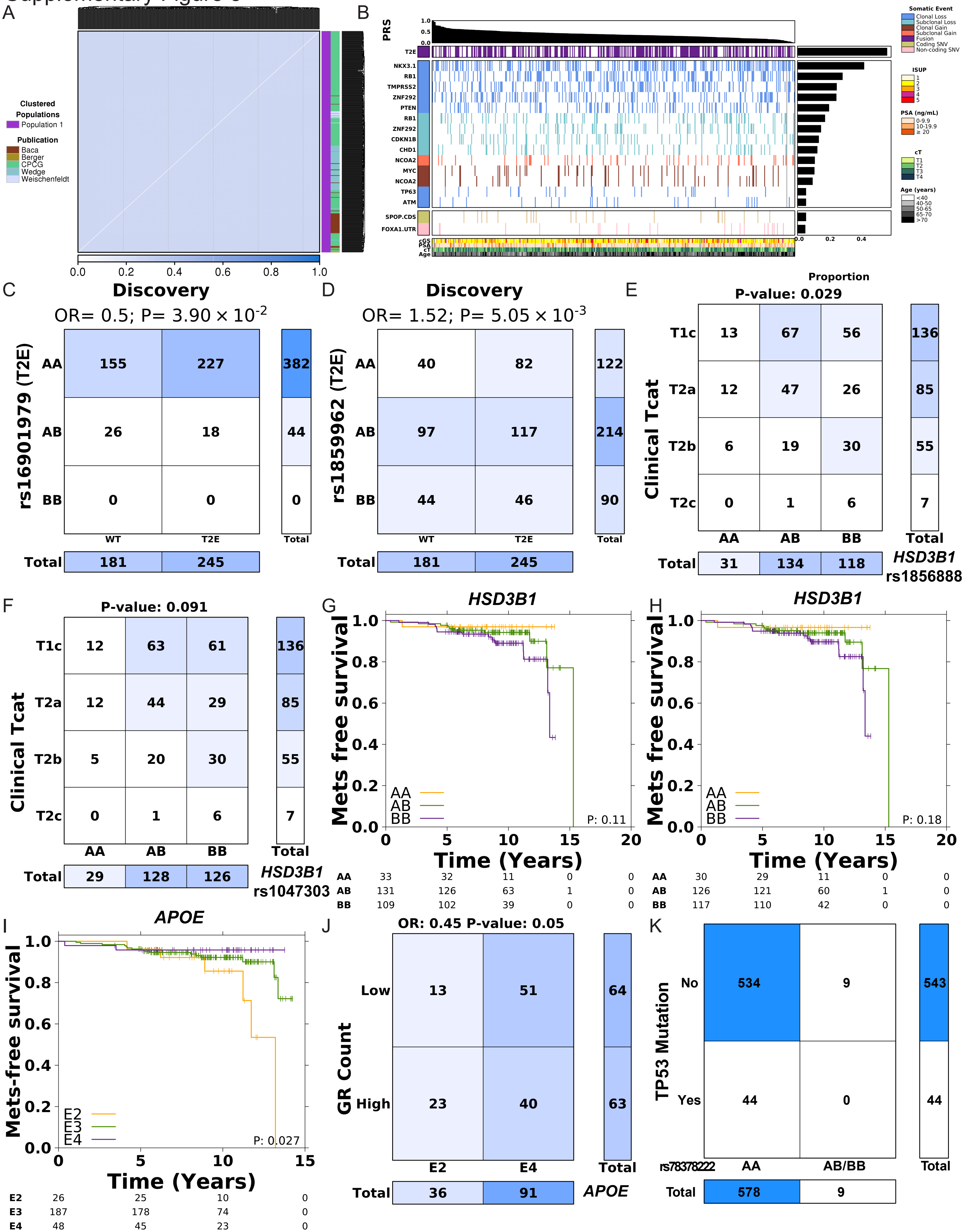

Supplementary Figure 8 | Cohort Characteristics and Risk dQTL Replication

A) Clustering using identity-by-state (IBS) as the distance metric showed no evidence of population substructure. The heatmap shows the identity-by-state values for all pairwise comparisons. The first covariate along the right shows the cluster provided by PLINK (v1.9). The second covariate indicates the original cohort the patient was published in. **B)** Landscape of somatic drivers in the discovery cohort. Somatic drivers are categorized as losses (blue), gains (red), SVs (purple), non-coding SNVs (pink) or coding SNVs (khaki). Barplot on the right shows the frequency of each driver in the discovery cohort. Covariate on the bottom indicates clinical characteristics of each patient including clinical ISUP Grade Group (ISUP), pre-treatment prostate serum antigen (PSA), clinical T category (cT) and age. Barplot on the top indicates the polygenic risk score (PRS), scaled between 0-1, for each patient. **C-D)** Contingency tables of rs16901979 **(C)** and rs1859962 **(D)** associated with T2E in the discovery cohort. **E-F)** Contingency tables of rs1856888 **(E)** and rs1047303 **(F)** associated with clinical T category. P-values from Fisher's exact test. **G-H)** Kaplan-Meier plots of rs1856888 **(G)** and rs1047303 **(H)** associated with metastasis-free survival (MFS). P-value from log-rank test. **I)** Kaplan-Meier plot of APOE genotypes associated with metastasis-free survival. P-value from log-rank test. **J)** Contingency table of *APOE2* and *APOE4* associated with GR count. OR and P-value from Fisher's Exact test. **K)** Contingency table showing stratification of individuals by genotype at rs78378222 and somatic alteration in *TP53*. A = reference allele, B = alternative allele

Supplementary Figure 9

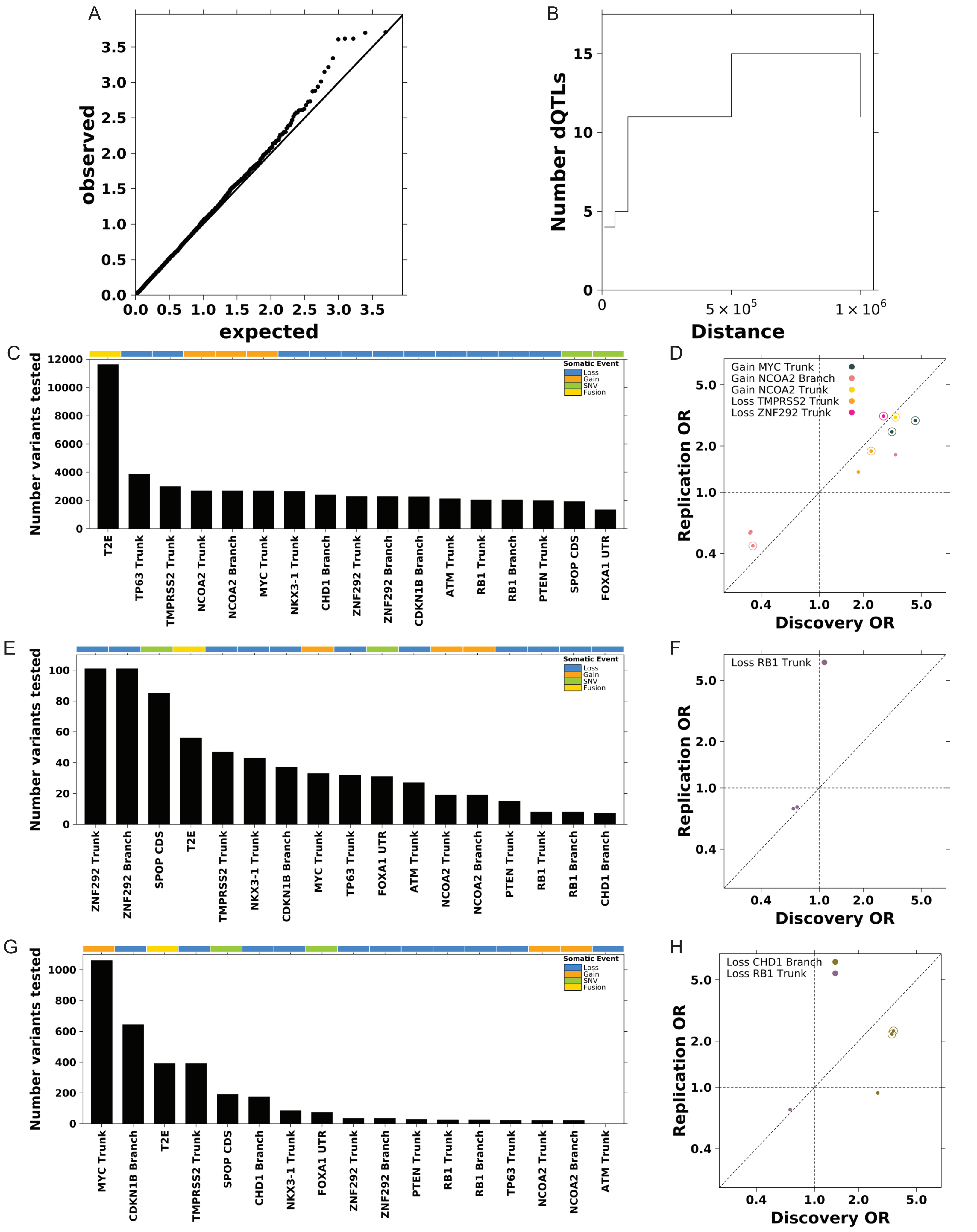

Supplementary Figure 9 | Local dQTLs Discovery

A) QQ plot of expected -$\log_{10}$ P-values *vs*. observed -$\log_{10}$ P-values for association of individual PRS SNPs with 17 drivers. **B)** Sensitivity plot showing number of discovered tag linear local dQTLs based on increasing distance from gene boundaries. **C)** Barplot shows the number of variants tested per somatic driver based on the linear definition of local dQTL. Covariate along the top indicates the type of somatic driver event. **D)** Comparison of ORs for linear local dQTLs with CNA drivers based on WGS profiling, x-axis and array profiling, y-axis. Horizontal and vertical dotted lines represent OR = 1 and diagonal lines represent y=x. Halo around points indicates Q < 0.1 in array-profiled cohort. **E)** Barplot shows number of variants tested per somatic driver based on spatial definition of local dQTL. Covariate along the top indicates the type of somatic driver. **F)** Comparison of ORs for spatial local dQTLs with CNA drivers based on WGS profiling, x-axis and array profiling, y-axis. Horizontal and vertical dotted lines represent OR = 1 and diagonal lines represent y=x. Halo around points indicates Q < 0.1 in array-profiled cohort. **G)** Barplot shows number of variants tested per somatic driver based on enhancer definition of local dQTL. Covariate along the top indicates the type of somatic driver. **H)** Comparison of ORs for enhancer local dQTLs with CNA drivers based on WGS profiling, x-axis and array profiling, y-axis. Horizontal and vertical dotted lines represent OR = 1 and diagonal lines represent y=x.

Supplementary Figure 10

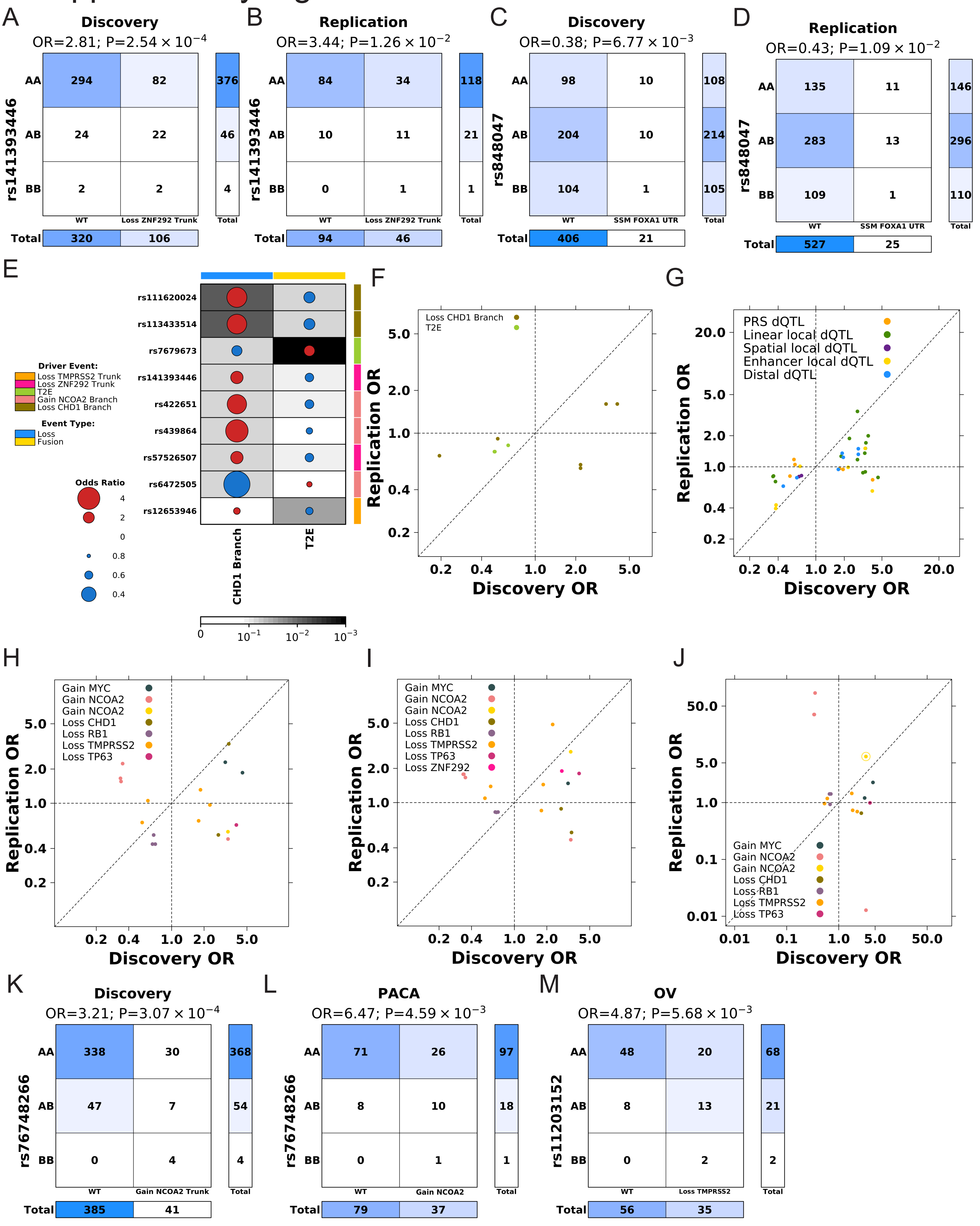

Supplementary Figure 10 | Replication of dQTLs

A-B) Contingency tables of rs141393446 associated with clonal loss of *ZNF292* in the discovery **(A)** and replication **(B)** cohorts. **C-D)** Contingency tables of rs848047 associated with SNVs in 3' UTR of *FOXA1* in discovery **(C)** and replication **(D)** cohorts. **E)** Replication of candidate distal dQTLs. **F)** Comparison of ORs in discovery, x-axis, *vs.* replication y-axis, cohorts of candidate distal dQTLs. Dot size and color indicate magnitude and direction of ORs between SNP, y-axis, and driver, x-axis. Covariate along the top indicates the type of somatic event. Covariate along the right indicates the associated somatic driver identified in discovery. **G)** Comparison of ORs in discovery, x-axis, and replication, y-axis, cohorts for 35 dQTLs. Dot color represents the strategy used to discover dQTLs. Horizontal and vertical dotted lines represent OR = 1 and diagonal lines represent y=x. **H-J)** Comparison of ORs in discovery, x-axis, *vs.* breast **(H)**, ovarian **(I)** or pancreatic **(J)** cancer, y-axis. Only testing dQTLs involving somatic drivers with recurrence rate >5% in each cancer type. Horizontal and vertical dotted lines represent OR = 1 and diagonal lines represent y=x. Halo indicates statistical significance (Q < 0.1). **K-M)** Contingency tables of rs76748266 associated with clonal loss of *NCOA2* in the discovery **(K)** and pancreatic **(L)** cohorts. **M)** Contingency table of rs11203152 associated with loss of *TMPRSS2* in ovarian cancer.

Supplementary Figure 11

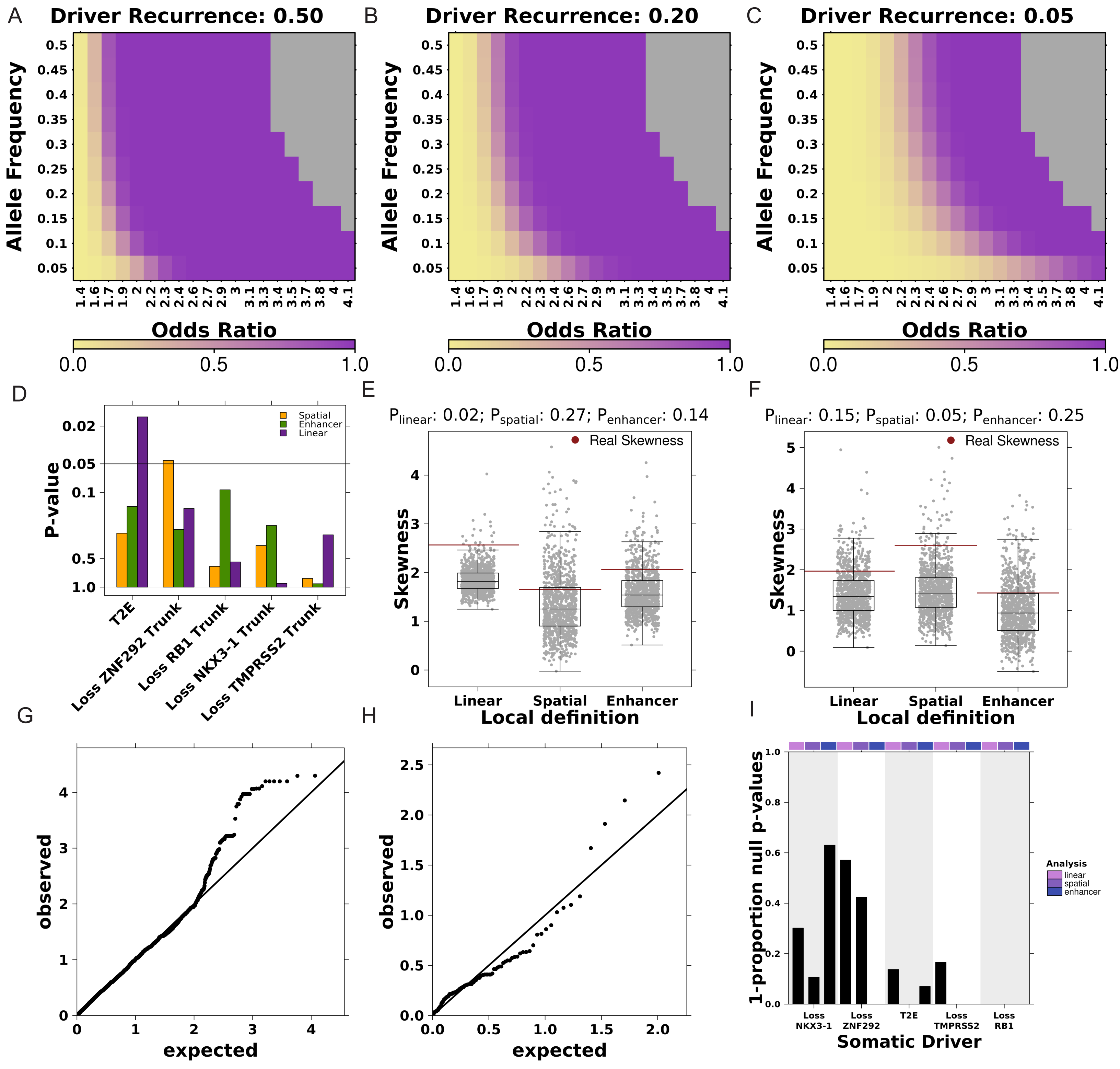

Supplementary Figure 11 | Enrichment of Sub-threshold dQTLs

A-C) Heatmaps displaying estimated power considering increasing ORs (x-axis) and allele frequencies (y-axis) for somatic driver recurrence = 0.50 **(A)**, 0.20 **(B)** and 0.05 **(C)**. Shading indicates estimated power with yellow = 0 and purple = 1. Grey indicates power could not be calculated. **D)** dQTL discovery P-value distributions are significantly skewed towards smaller P-values. The P-value skew for each dQTL discovery for the top five most recurrent somatic drivers was compared to an empirically generated null distribution (iterations=1,000) and a P-value calculated as the number of null iterations with skew > real skew. Barplot shows the P-value from this permutation analysis. Horizontal line indicates P = 0.05 and colors represent the dQTL discovery approach. **E)** Null skew distribution for T2E dQTL discovery from 1,000 iterations. Horizontal red lines represent real skew values for each dQTL approach. P-values along the top represent the number of null iterations with skew > real skew divided by the number of null iterations. Boxplot represents median, 0.25 and 0.75 quantiles with whiskers at 1.5x interquartile range. **F)** Null skew distribution of clonal loss of *ZNF292*. **G-H)** Q-Q plots of T2E linear local dQTLs **(G)** and clonal loss of *ZNF292* spatial local dQTLs **(H)**. **I)** Barplot of 1 – the estimated proportion of null P-values (y-axis) in linear, spatial or enhancer dQTL discovery for the top five most recurrent somatic events. The estimated proportion of null P-values of spatial dQTLs associated with clonal loss of *RB1* could not be tested due to too few SNPs.

Supplementary Figure 12

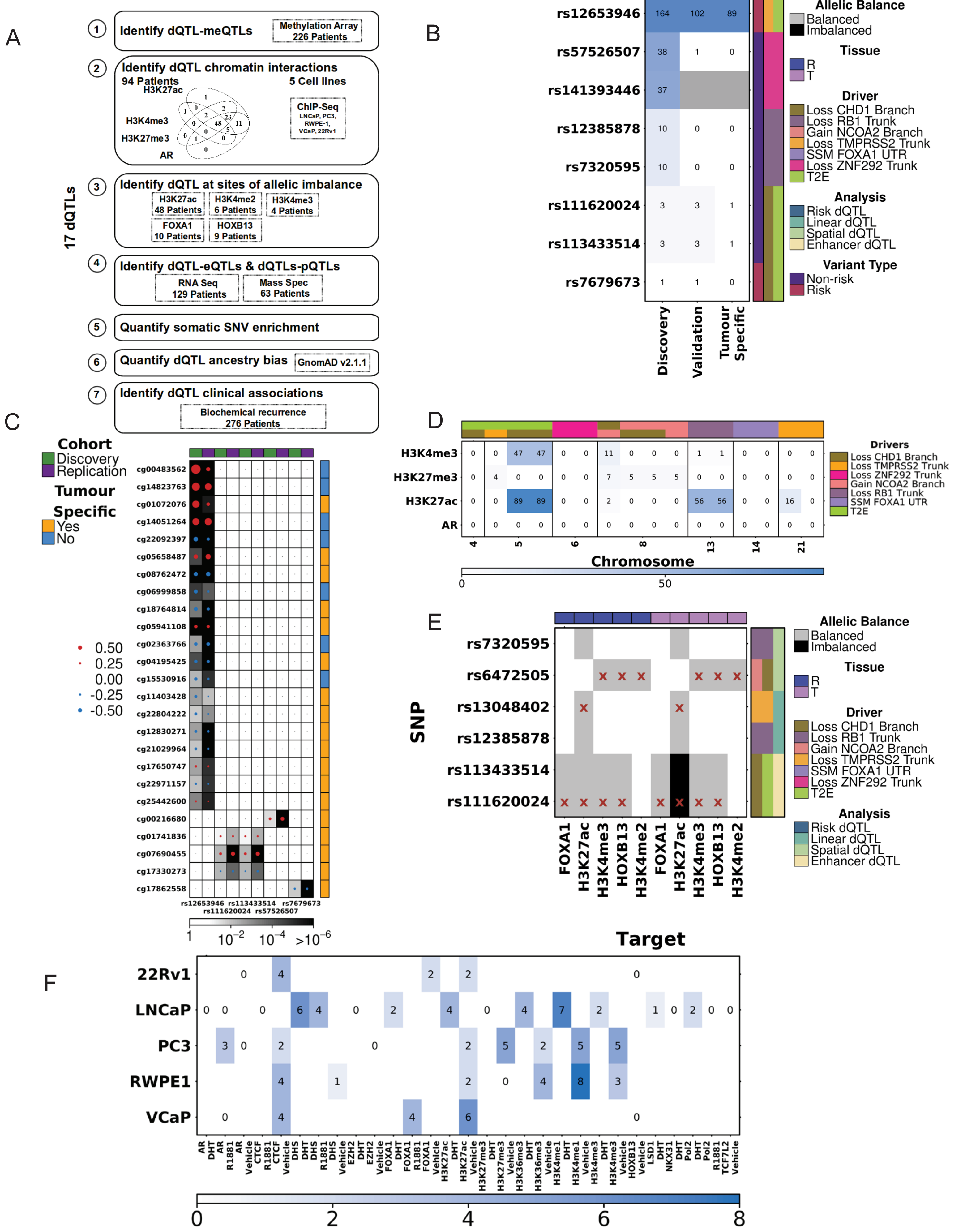

Supplementary Figure 12 | Molecular Characterization of dQTLs

A) Schematic of characterization of dQTLs. **B)** dQTLs (rows) and the number of differentially methylated tumor regions in discovery and replication cohorts, and the number that were tumor-specific. Right covariate indicates risk variants and somatic drivers. **C)** Summary of dQTL-meQTLs. Circle size and color represents the magnitude and direction of meQTL in discovery and replication cohorts. Background shading indicates false discovery rate. Covariate along the top indicates discovery or replication cohort. Covariate along the right indicates if the meQTL was identified as tumor specific. Highly correlated probes (Spearman's r > 0.80) are summarized by a single probe and only the top 20 most correlated probes for rs12653946 are plotted. **D)** Overlap of dQTLs, x-axis, with histone modifications and AR binding in primary patient samples, y-axis. Shading indicates the number of patients with overlap. Covariate along the top indicates the somatic driver(s) each SNP is associated with. **E)** dQTL variants (rows) and associated histone modification and transcription factor binding sites (columns). Grey shading indicates overlap with allelic balanced ChIP-Seq peaks; black indicates overlap with allelic imbalanced ChIP-Seq peak. Red X indicates overlapping SNP is tag SNP. Top covariate indicates tissue while right covariate indicates risk variants and somatic drivers. **F)** Overlap of dQTLs with histone modification and transcription factor binding sites, x-axis, in five prostate cell lines, y-axis. Shading indicates the number of dQTLs that overlap each target in each cell line. X-axis labels give the target and treatment.

Supplementary Figure 13

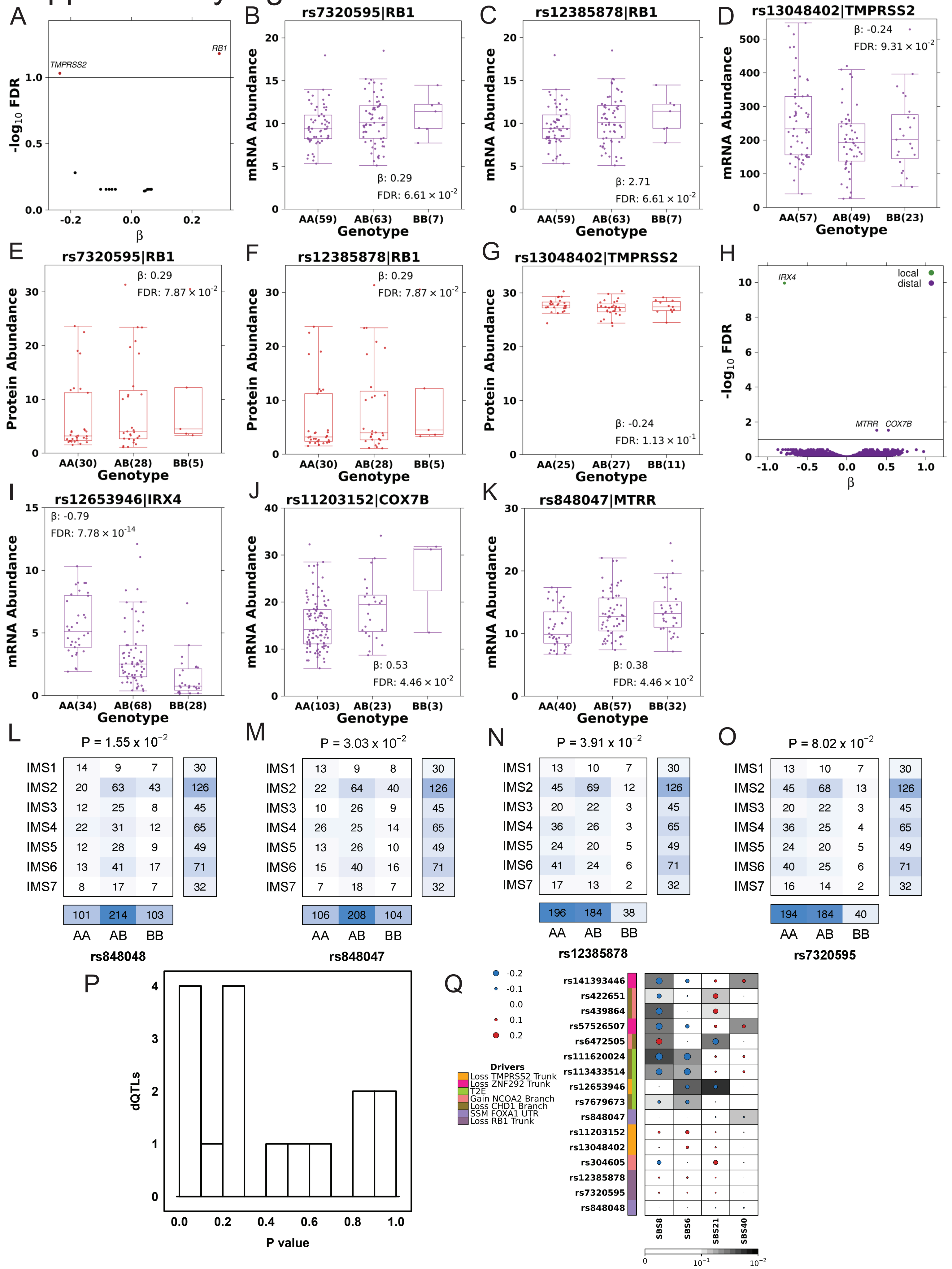

Supplementary Figure 13 | Association of dQTL Risk SNPs with eQTL and IMS

A) Volcano plot of candidate eQTLs – dQTL variant associated with mRNA abundance of associated driver gene. Y-axis shows $\log_{10}$ false discovery rate while x-axis shows effect size of association. Horizontal line indicates Q = 0.1 and red dots indicates a significant association ($Q < 0.1$). **B-G)** dQTLs are associated with mRNA **(B-D)** and protein **(E-G)** abundance changes of associated driver gene. Boxplot shows mRNA (purple) or protein (red) abundance for gene in title stratified by genotype, x-axis, of the SNP indicated in the title. Statistics are from inverse rank-normalized linear regression model correcting for the first five principal components and age. The number of samples with each genotype is indicated in parenthesis next to the genotype along the x-axis. Boxplot represents median, 0.25 and 0.75 quantiles with whiskers at 1.5x interquartile range. **H)** Volcano plot of local (green) and distal (purple) eQTLs. **I)** One local eQTL identified between rs12653946 and *IRX4*. **J-K)** Two distal eQTLs identified: rs11203152-*COX7B* and rs848047-*MTRR.* **L-O)** Four risk SNPs significantly associated with IMS subtypes ($P < 0.1$; X^2 test) **P)** P-value histogram of all dQTL risk SNPs associated with IMS subtypes determined using X^2 test. **Q)** Dotmap showing the association between 16 unique dQTL variants and four SBS signatures. Dot size and color indicates effect size magnitude and direction from linear model correcting for the first five genetic principal components, age and SNV burden. Background shading indicates nominal p-value. Covariate on the left indicates the somatic driver each variant is associated with.

Supplementary Figure 14

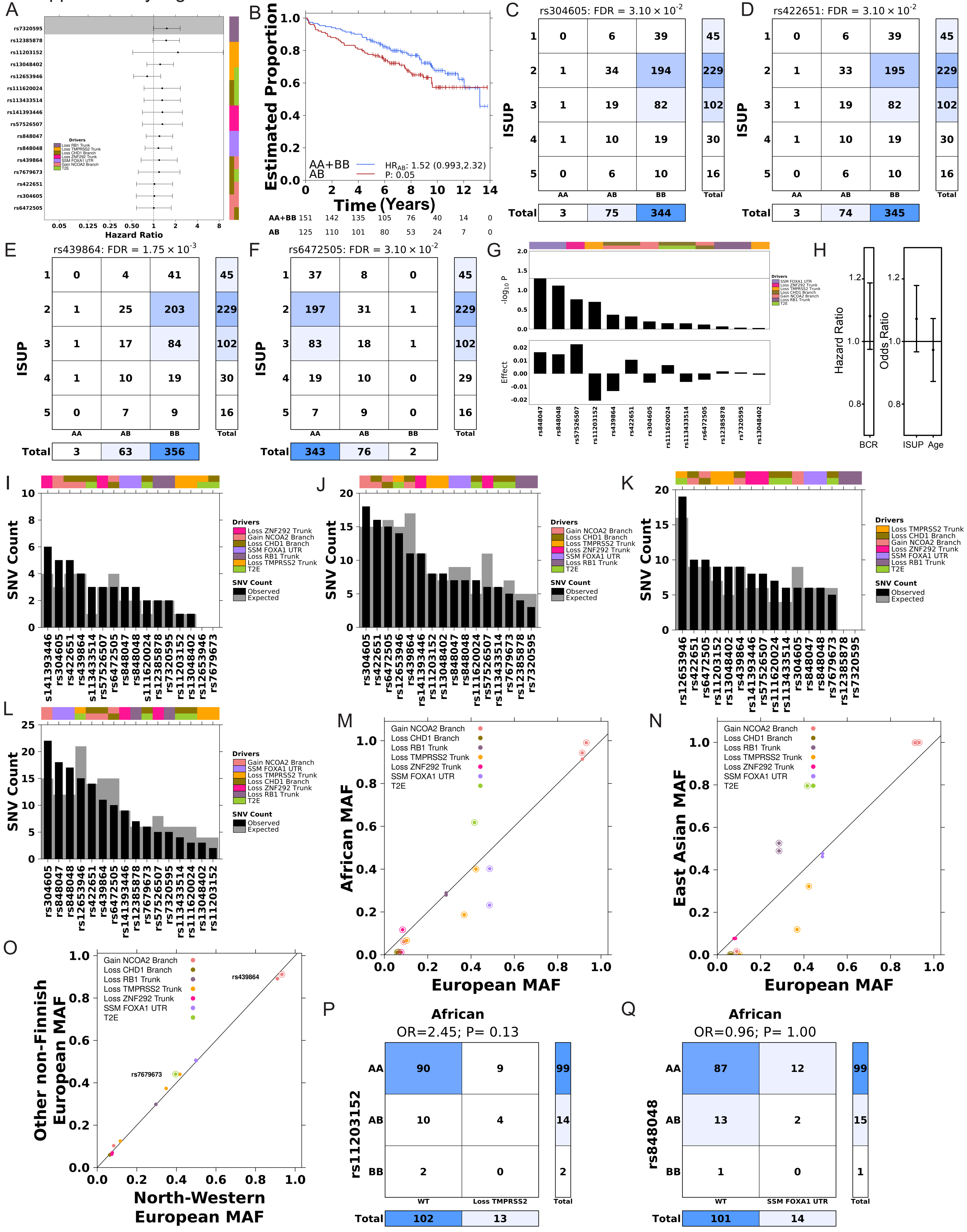

Supplementary Figure 14 | Clinical Characterization of dQTLs

A) Forest plot showing Hazard Ratios, x-axis, from survival analysis of dQTLs, y-axis, with biochemical recurrence. Error bars represent 95% confidence intervals. Vertical dotted line represents HR = 1. Background shading indicates $P < 0.05$ and covariate on the right indicates the somatic driver event the SNP is associated with. **B)** Kaplan-Meier plot of rs7320595 associated with biochemical recurrence. **C-F)** Contingency tables of association between rs304605 **(C)**, rs422651 **(D)**, rs439864 **(E)** and rs6472505 **(F)** and ISUP Grade Group. FDR from ordinal linear regression. **G)** Barplot shows effect size and P-value from prostate cancer GWAS(16) for 13 non-risk dQTLs, x-axis, with summary statistics from GWAS. Horizontal line indicates P= 0.05. Covariate along the top indicates an associated somatic driver. **H)** Forest plot shows the hazard ratio (left) and odds ratio (right) for the association between dQTL burden and biochemical recurrence (BCR), ISUP grade group and age at treatment from a CoxPH and logistic model, respectively, and correcting for the first two genetic principal components. Error bars show 95% confidence intervals. **I-L)** Number of somatic SNVs within ±10 kbp, y-axis, of each dQTL, x-axis, in prostate **(I)**, breast **(J)**, ovarian **(K)** and pancreatic **(L)** cancer. Background shading indicates the number of proximal somatic SNVs expected by chance. Covariate along the top indicates the somatic driver event each SNP is associated with. **M-O)** Comparison of allelic frequencies for 16 dQTLs in European, x-axis *vs.* African, y-axis, populations **(M)**, European *vs.* East Asian populations **(N)** or within European populations **(O)**. Halo indicates SNP has significantly different allele frequencies across populations. **P)** Contingency table of rs11203152 associated with loss of *TMPRSS2* in 115 African men. **Q)** Contingency table of rs848048 associated with SNVs in *FOXA1* UTR in 115 African men.
